# Complex Interactions between Genes and Social Environment Cause Phenotypes Associated with Autism Spectrum Disorders in Mice

**DOI:** 10.1523/ENEURO.0124-20.2020

**Published:** 2020-08-05

**Authors:** Monika Sledziowska, Shireene Kalbassi, Stéphane J. Baudouin

**Affiliations:** School of Biosciences, Cardiff University, Cardiff CF10 3AX, United Kingdom

**Keywords:** autism spectrum disorders, motor learning, mouse behavior, social environment

## Abstract

The etiology of autism spectrum disorders (ASDs) is a complex combination of genetic and environmental factors. Neuroligin3, a synaptic adhesion protein, and cytoplasmic *FMR1* interacting protein 1 (CYFIP1), a regulator of protein translation and actin polymerization, are two proteins associated with ASDs that interact in neurons *in vivo*. Here, we investigated the role of the Neuroligin3/CYFIP1 pathway in behavioral functioning and synapse formation in mice and found that it contributes to motor learning and synapse formation in males. Similar investigation in female mice revealed an absence of such phenotypes, suggesting that females are protected against mutations affecting this pathway. Previously, we showed that the social environment influences the behavior of male mice. We extended this finding and found that the transcriptome of wild-type mice housed with their mutant littermates, lacking Neuroligin3, differed from the transcriptome of wild-type mice housed together. Altogether, these results identify the role of the Neuroligin3/CYFIP1 pathway in male mouse behavior and highlight its sensitivity to social environment.

## Significance Statement

The causes of autism spectrum disorders (ASDs) remain elusive. In this study, we investigate the combined effect of mutations in two genes associated with ASDs, *Nlgn3* and *Cyfip1*, and the effect of the social environment on phenotypes relevant for ASDs. We show that when both mutations are present the behavior can be restored, emphasizing the importance of considering gene interactions. We also show sex differences in behavior, suggesting that female subjects should be included in the studies of ASDs. We show that wild-type animals can exhibit phenotypes associated with ASDs as a result of being housed with their mutant littermates, highlighting the necessity to re-evaluate the use of wild-type animals as controls to define phenotypic traits of mouse models.

## Introduction

Autism spectrum disorders (ASDs) are characterized by behavioral manifestations, primarily social communication deficits and stereotyped behavior, frequently accompanied by a wide range of secondary comorbid clinical symptoms (American Psychiatric Association, 2012). The etiology of ASDs is far from being fully understood but is likely to be a complex interaction between specific genetic mutations and environmental factors triggering the emergence of a constellation of behavioral symptoms. Understanding the relationship between the different causes of the disorder and the behavioral manifestation is key for the development of targeted treatments.

Among the genes associated with ASDs is the X-linked gene *NLGN3* (Jamain et al., 2003; Talebizadeh et al., 2006; Glessner et al., 2009; Yasuda et al., 2011; Sanders et al., 2015; Gumus, 2019), coding for the synaptic adhesion molecule Neuroligin3 (Chih et al., 2004; Varoqueaux et al., 2006) and cytoplasmic *FMR1* interacting protein 1 (*CYFIP1*; Wang et al., 2009; van der Zwaag et al., 2009; Toma et al., 2014; Picinelli et al., 2016), and coding for a protein involved in actin polymerization regulation (Chen et al., 2010) and the regulation of protein translation (Napoli et al., 2008). In male mice, the lack of *Nlgn3* is associated with impaired social behavior (Radyushkin et al., 2009; Fischer and Hammerschmidt, 2011; Kalbassi et al., 2017; Bariselli et al., 2018), hyperactivity (Radyushkin et al., 2009), and subtle changes in the rate of motor learning (Baudouin, 2014; Rothwell et al., 2014). In mice, *Cyfip1* haploinsufficiency is also associated with reduced interest in social odors, hypoactivity, and motor learning impairment (Bozdagi et al., 2012; Bachmann et al., 2019). Interestingly, the cytoplasmic tail of Neuroligin3 contains a WAVE regulatory complex interactor receptor sequence, which can bind CYFIP1 (Chen et al., 2014; Bachmann et al., 2019), an interaction that was recently confirmed in neurons *in vivo* (Bachmann et al., 2019). The interaction between Neuroligin3 and CYFIP1 suggests that they are part of the same molecular pathway and that disruption of this pathway can cause some of the phenotypes associated with ASDs.

At the subcellular level, in neurons, both proteins have been associated with the formation and elimination of synapses. Triple knockout of *Nlgn3* as well as two other members of this gene family, *Nlgn1* and *Nlgn2*, results in a reduction of synaptic contacts *in vivo* and lower dendritic spine number *in vitro* (Chih et al., 2004; Varoqueaux et al., 2006), supporting the idea that *Nlgn3* might play a role in synaptic development and maintenance. *Cyfip1* haploinsufficiency is associated with a decrease in spine density in the motor cortex and olfactory bulb in male mice (Abekhoukh et al., 2017; Bachmann et al., 2019) as well as an increased number of filamentous spines (De Rubeis et al., 2013; Pathania et al., 2014; Abekhoukh et al., 2017; Davenport et al., 2019). This is potentially explained by the increased rate of formation and elimination of dendritic spines seen in these animals (Bachmann et al., 2019). Importantly, the alteration of dendritic spine density is one of the few cellular phenotypes of ASDs observed both in mouse models and in human patients (Martínez-Cerdeño, 2017). The overall aim of our study was to investigate how the interaction between *Nlgn3* and *Cyfip1* can influence ASD-relevant phenotypes in mice, from behavioral alterations to changes at the cellular level.

The complex genotype–phenotype relationships are further shaped by factors such as sex of the subjects and their social environment. For example, only females with the complete deletion of *Nlgn3* showed deficits in behavior (Kalbassi et al., 2017). The social environment might also be an environmental factor triggering ASD-related phenotypes. Male mice lacking *Nlgn3* and wild-type (WT) mice influence each other’s behavior, an effect attributed to the role of *Nlgn3* in controlling social dominance. Housing mice carrying the 16p11.2 microdeletion, another model of ASD, with littermates of a different genotype was also shown to influence the vocalization during courtship (Yang et al., 2015). In addition to investigating the role of interaction between *Nlgn3* and *Cyfip1* in controlling behavioral phenotypes, the secondary aims of this study were to determine the role of sex in the phenotypic outcome of mutations in *Nlgn3* and *Cyfip1* and the effect of social environment on mRNA expression in these mice.

## Materials and Methods

### Animals

All procedures were conducted in accordance with the Animals (Scientific Procedure) Act 1986 (amended in 2012). Mice were kept on a 12 h light/dark cycle with free access to food and water, in groups of two to five in a cage. All behaviors were assessed during the light phase of the light/dark cycle. All mice were tested as adults, P60– P70 at the start of testing. Mice were handled for at least 2 days before any procedure. The mice were habituated to the room where the behavioral assessment was taking place for 30 min before commencing any procedure.

*Nlgn3^y/−^, Nlgn3*^+/−^ (Tanaka et al., 2010) and *Cyfip1*^+/−^ (EUCOMM) mice were crossed with mice containing *Thy-EGFP* transgene (stock # 007788, The Jackson Laboratory; Feng et al., 2000) to obtain the following male mice: *Nlgn3^y/−^Thy1-EGFP*, *Cyfip1*^+/−^*Thy1-EGFP*, *Nlgn3^y/−^Cyfip1*^+/−^*Thy1-EGFP*, and *Thy1-EGFP*; and the following female mice: *Nlgn3*^+/−^*Thy1-EGFP*, *Cyfip1*^+/−^*Thy1-EGFP*, *Nlgn3*^+/−^*Cyfip1*^+/−^*Thy1-EGFP*, and *Thy1-EGFP*. Thus, all of the mice used in the behavioral experiments and used to investigate the dendritic spine density were littermates. A proportion of the mice used in the behavioral experiments had the *Thy-EGFP* transgene. The lack of an effect of the transgene on the behavioral outcomes was confirmed by repeating every statistical analysis with the presence of the transgene as a variable. There were no significant differences in any of the behavioral outcomes between the mice with and without the transgene.

In the first RNA experiment, an additional cohort of wild-type animals was bred and included in the analysis. In the second RNA experiment, a cohort of wild-type animals was bred where the parents came from the *Nlgn3* colony, ensuring that the mutant and wild-type mice shared parents.

### Activity in open field

The spontaneous activity of mice was recorded. Mice were tested on 2 consecutive days. During the test, the mice were individually placed in an open field arena (40 × 20 cm) and were allowed to explore. The test was conducted in the dark; however, the bottom of the arena was illuminated by an infrared lamp to allow tracking of the mice. The movement of the mice was recorded using a video camera above the arena. The traces were recorded and quantified in EthoVision XT (Noldus).

### Rotarod

Motor learning of the mice was assessed using a rotarod (Jones and Roberts, 1968). Latency to fall off the rod was assessed for 3 days in a row, with 10 subsequent 5 min trials each day. During a trial, mice were placed on the rod. The rotarod was then switched on and accelerated from 4 to 40 rpm over the course of 5 min. The mice were allowed to walk on the rod until they fell off, gripped to the rod and the rod made a full revolution, or 5 min have passed. Falling off or gripping the rod was interpreted as an inability to cope with the task and signaled the end of the trial. Latency to fall was measured using a stopwatch (Casio). After each trial, the mice were allowed to rest for 5 min at the bottom of the apparatus.

### Social odor interest

Social odors originated from a cage of three to four WT mice that were maintained with the same home cage bedding for a week to allow for the concentration of odorants present in the urine. For some of the trials, the cage also contained a maximum of one *Nlgn3^y/−^Cyfip1*^+/−^ or *Nlgn3*^+/−^*Cyfip1*^+/−^ mouse. Before the trial, a cotton bud was wiped across the bottom of the home cage in a zig-zag fashion to obtain the social odor cue. Mice were placed in the experimental arena and were allowed to habituate for 2 min. Mice were exposed to a clean cotton bud for 2 min, which was then swapped for a new clean cotton bud, and mice were allowed to interact with it for another 2 min. Next, the mice were exposed to the cotton bud with the odor cue for 2 min, which was finally swapped for a new cotton bud with an olfactory cue for another 2 min. Male mice were exposed to olfactory cues originating from a cage of male mice, while female mice were exposed to olfactory cues originating from a cage of female mice. The trials were recorded with a video camera placed above the experimental arena. Time spent sniffing the cotton bud was scored manually, blinded to the genotype.

### Courtship vocalization

Female mice in estrus were identified using vaginal lavage, followed by cytological staining (Giemsa solution, Polysciences) and visual assessment. Male mice were habituated to the experimental arena (40 × 20 cm) for 3 min. Next, an unfamiliar female mouse in estrus was added to the arena, and the mice were allowed to interact freely for 3 min. Ultrasonic vocalizations (USVs) between 40 and 250 Hz produced by the male mice were recoded using a preamplifier (UltraSoundGate 416 H, Avisoft Bioacoustics) connected to a microphone (UltraSoundGate CM16, Avisoft Bioacoustics). The total number of USVs and their duration was analyzed using SASLabPro (Avisoft Bioacoustics).

### Histology

Dendritic spine quantification was conducted in the motor and visual cortices of *Nlgn3^y/−^Cyfip1*^+/−^, *Nlgn3^y/−^*, *Cyfip1*^+/−^ males and their WT littermates, as well as of *Nlgn3*^+/−^*Cyfip1*^+/−^, *Nlgn3*^+/−^, and *Cyfip1*^+/−^ females and their WT littermates, all of which also expressed EGFP under the *Thy-1* promoter. Mice were anesthetized with Euthatal and perfused with 4% paraformaldehyde in 0.1 m PBS. The entire brain was dissected out and postfixed overnight in the 4% paraformaldehyde in 0.1 m PBS, kept in 30% sucrose solution until saturated and stored at −80°C. The brains were cut coronally into 50 μm sections on a cryostat (Leica Biosystems) and immediately mounted on glass slides. The regions of interest were identified using a mouse brain atlas (Paxinos and Franklin, 2004), and *Z*-stack images spaced 0.5 mm apart were acquired on a Zeiss LSM700 upright confocal microscope (Carl Zeiss), using a 40 water-immersion lens. The images were reconstructed into two dimensions using *Z*-stack maximum intensity projection in ImageJ (NIH). The images were analyzed by an experimenter blinded to the genotype of the animals. Spines were identified manually and counted on a 20- to 250-μm-long stretch of a dendrite, with a minimum of 24 dendrites from four mice, per condition. Spine density was calculated as the number of spines per 10 μm of a dendrite.

### RNA sequencing

All procedures were conducted in RNAase-free conditions. Mice were culled by cervical dislocation, their brains were extracted, and relevant brain regions were dissected following a mouse brain atlas (Paxinos and Franklin, 2004). The samples were immediately snap frozen in liquid nitrogen and stored at −80°C. Invitrogen TRIzol Reagent (Thermo Fisher Scientific) was added to the samples, at 1 ml per 50–100 mg of tissue. Tissue was homogenized and incubated at room temperature for 5 min. Samples were transferred to Invitrogen Phrasemaker Tubes (Thermo Fisher Scientific), and 0.2 ml of chloroform was added. The samples were shaken for 15 s, incubated at room temperature for 15 min, and centrifuged for 5 min at 14,000 × *g*, at 4°C. The RNA-containing upper phase was mixed with 0.5 ml of isopropanol, and the samples were incubated for 1 h at −80°C. Following the incubation, the samples were centrifuged for 10 min at 10,000 × *g*. The supernatant was removed, and the pellet was washed using 75% EtOH. The pellet was dissolved in RNA-free water and treated with DNase (QIAGEN) as per manufacturer instructions.

For reverse transcription, 1250 ng of RNA was incubated with 1 μl of random primers (Promega) and 1 μl of deoxyribonucleotide triphosphates (dNTPs; 10 mm, Promega) for 5 min, at 65°C and for 1 min on ice. Then 4 μl of buffer (Thermo Fisher Scientific), 1 μl dithiothreitol (0.1 m, Thermo Fisher Scientific), 1 μl Rnasin plus (Promega), and 1 μl of superscript III reverse transcriptase (Thermo Fisher Scientific) were added, and the mix was incubated for 5 min at room temperature and then for 2 h at 50°C. The samples were then incubated for 10 min at 70°C.

Quality control of the RNA samples was confirmed by Tape Station and Qubit. The library was prepared according to manufacturer instruction (Illumina TruSeq). Total RNA was purified to remove ribosomal and non-mRNA with magnetic beads. mRNA was then transferred for first-strand cDNA synthesis with superscript II. Next, the second strand of the cDNA was synthesized, and the template was eradicated. Adapters were ligated to the cDNA. The cDNA was then amplified to enrich the libraries and tested on a DNA chip for quality control, and the library size was normalized. The RNA sequencing was performed according to manufacturer instructions using the Illumina NextSeq500 System in 1 × 75 bp cartridge. A strand of cDNA was bound to a docking site, and fluorescent dNTPs were added one at a time.

The sequences were trimmed with Trimmomatic (Bolger et al., 2014) and assessed for quality with FastQC. STAR was used to map the reads onto the Mouse Genome Assembly GRCm38. Transcripts were assigned using Feature Counts (Dobin et al., 2013; Liao et al., 2013). Downstream analysis was performed in R version 2.6.2 (R Core Team, 2019). The DESeq2 package was used for differential gene expression (Love et al., 2014). The relative expression of genes was assessed in pairwise fashion to include all housing and genotype conditions. The values were normalized using the implementation of variance-stabilizing transformation from the DESeq2 package. Principal component analysis of the top 100 genes with the greatest fold expression differences was conducted using the R function procomp. Weighted gene correlation network analysis (Langfelder and Horvath, 2008) was performed on normalized data, with a power of 5 and a minimum module size of 200.

### Statistical analysis

The data analysis was conducted using R software, version 3.6.2. (R Core Team, 2019) or when an appropriate package was not available using Graph Pad Prism version 8.3.1. The normality of raw data or residuals was checked using the Shapiro–Wilk test as well as visual inspection of Q-Q plots and histograms. The homogeneity of variance was checked using Levene’s test or visual inspection of a plot of residuals versus plotted values, depending on the data type. If the data were deemed to violate the assumption of normality or homogeneity of variance, an appropriate nonparametric test was used. The nonparametric mixed ANOVA was conducted according to Noguchi et al. (2012). The details of normality assessment, the tests used, and the number of samples per group can be found in the statistics table in Extended Data Figure 1-2.

**Figure 1.**
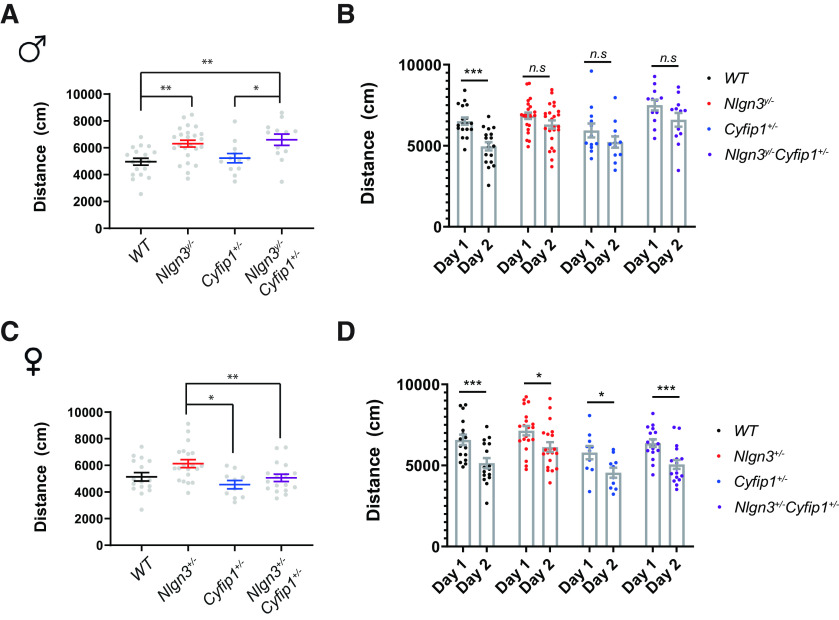
The effect of *Nlgn3* deletion and *Cyfip1* haploinsufficiency on exploratory behavior. ***A***, Distance traveled in the open field by the *Nlgn3^y/-^Cyfip1*^+/−^ male mice and their littermates. ***B***, Habituation to the novel environment in the *Nlgn3^y/−^Cyfip1*^+/−^ male mice and their littermates in the open field. ***C***, Distance traveled in the open field in by the *Nlgn3*^+/−^*Cyfip1*^+/−^ female mice and their littermates. ***D***, Habituation to the novel environment in the *Nlgn3*^+/−^*Cyfip1*^+/−^ female mice and their littermates in the open field. *p <* *0.05, **p <* *0.01, ***p <* *0.001, n.s., not significant. Comparison of the time spent in the center of the open field arena and sex is available in Extended Data Figure 1-1. Details of statistical analysis are available in Extended Data Figure 1-2.

10.1523/ENEURO.0124-20.2020.f1-1 Figure 1-1 The effect of *Nlgn3* deletion and *Cyfip1* haploinsufficiency on anxiety. ***A***, Time spent in the center of the open field arena for *Nlgn3^y/−^Cyfip1*^+/–^ male mice and their littermates. ***B***, Time spent in the center of the open field arena for *Nlgn3*^+/–^*Cyfip1*^+/–^ female mice and their littermates. ***C***, Comparison of the distance traveled in the open field between WT and *Cyfip1*^+/–^ males and females. ***D***, Comparison of the time spent in the center of the open field between WT and *Cyfip1*^+/–^ males and females. Download Figure 1-1, EPS file.

**Figure 2.**
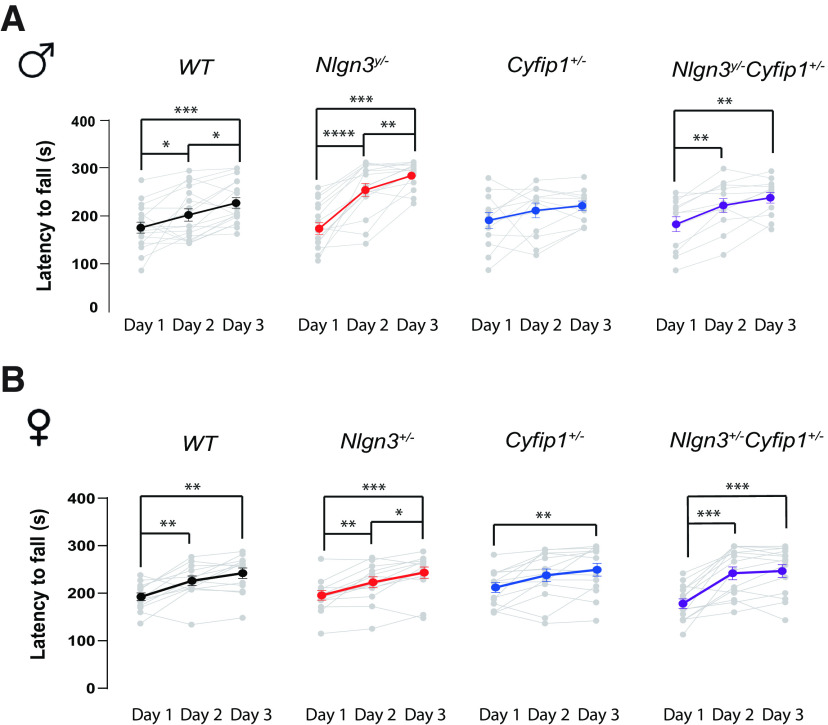
The effect of *Nlgn3* deletion and *Cyfip1* haploinsufficiency on motor learning. ***A***, Latency to fall off the rotarod in the *Nlgn3^y/-^Cyfip1*^+/−^ male mice and their littermates. ***B***, Latency to fall off the rotarod in the *Nlgn3*^+/−^*Cyfip1*^+/−^ female mice and their littermates. *p <* *0.05, **p <* *0.01, ***p <* *0.001, ****p < 0.0001. Data for the comparison of individual trials and sex are available in Extended Data Figure 2-1.

10.1523/ENEURO.0124-20.2020.f1-2 Figure 1-2 Statistical table. Download Figure 1-2, DOCX file.

10.1523/ENEURO.0124-20.2020.f2-1 Figure 2-1 The effect of *Nlgn3* deletion and *Cyfip1* haploinsufficiency on motor learning across trials. ***A***, Latency to fall off the rotarod in the *Nlgn3^y/−^Cyfip1*^+/–^ male mice and their littermates across trials. ***B***, Latency to fall off the rotarod in the *Nlgn3*^+/–^*Cyfip1*^+/–^ female mice and their littermates across trials. ***C***, Comparison of the latency to fall off the rotarod between WT and *Cyfip1*^+/–^ males and females. Download Figure 2-1, EPS file.

## Results

### *Nlgn3^y/−^Cyfip1*^+/−^ mice phenocopy the enhanced exploratory behavior of *Nlgn3^y/−^* males

We generated *Nlgn3^y/−^Cyfip1*^+/−^ double-mutant mice to investigate the effect of the *Nlgn3/Cyfip1* pathway on phenotypes associated with autism: changes in behavior and dendritic spine density. In the first instance, we investigated the behavior of the mutant mice.

As previously reported *Nlgn3^y/−^* males (*n *=* *24; day 2: mean = 6307.63 cm, SE = 256.25 cm) were hyperactive in the open field compared with their WT littermates (*n *=* *18; day 2: mean = 4962.81 cm, SE = 258.24 cm; one-way ANOVA, main effect of genotype: *F*_(3,62)_ = 6.55, *P* = < 0.001, Tukey’s HSD: *t*_(1,62)_ = 3.47, *p *<* *0.01; Fig. 1*A*). Similarly, *Nlgn3^y/−^Cyfip1*^+/−^ mice (*n *=* *12; day 2: mean = 6597.82 cm, SE = 420.93 cm) traveled farther in the open field than their WT littermates (Tukey’s HSD: *t*_(1,62)_ = 3.53, *p* < 0.01). The *Nlgn3^y/−^Cyfip1*^+/−^ mice were also hyperactive in relation to their *Cyfip1*^+/−^ littermates (*n *=* *12; day 2: mean = 5227.60 cm, SE = 345.70 cm*; t*_(1,62)_ = 2.7, *p *<* *0.05). However, there was no evidence of a more pronounced phenotype in the *Nlgn3^y/−^Cyfip1*^+/−^ mice as they did not significantly differ from the *Nlgn3^y/−^*single-mutant mice. The same pattern of results emerged when the effect of genotype on the distance traveled was considered across the two days of testing (mixed-model ANOVA, main effect of genotype: *F*_(3,62)_ = 6.45, *p *<* *0.001, main effect of day: *F*_(1,62)_ = 35.54, *p *<* *0.001; Tukey’s HSD: WT vs *Nlgn3^y/−^ t*_(1,62)_ = −2.70, *p *<* *0.05, WT vs *Nlgn3^y/−^Cyfip1*^+/−^ *t*_(1,62)_ = −3.53, *p *<* *0.01, *Cyfip1*^+/−^ vs *Nlgn3^y/−^ t*_(1,62)_ = −2.82, *p *<* *0.05, *Cyfip1*^+/−^ vs *Nlgn3^y/−^Cyfip1*^+/−^ *t*_(1,62)_ = −3.61, *p *<* *0.01). The hyperactivity in these mice may arise from a more pronounced response to a novel environment. To investigate this possibility, we considered the habituation pattern between the first and second day of testing. A significant decrease in the distance traveled between the 2 days was only observed in the WT males (Tukey’s HSD: *t*_(2,62)_ = −5.30, *p *<* *0.001; Fig. 1*B*), suggesting a habituation deficit in the *Nlgn3^y/−^*, *Cyfip1*^+/−^, as well as *Nlgn3^y/−^Cyfip1*^+/−^ mice. The phenotype was unlikely to occur because of differences in anxiety levels as there were no differences between genotypes in the time spent in the center of the open field (Extended Data Fig. 1-1*A*).

Sex is known to impact on the rate of diagnosis of ASDs (Werling and Geschwind, 2013); thus, we aimed to investigate potential sex differences in exploratory behavior. We tested *Nlgn3*^+/−^*Cyfip1*^+/−^ (*n = *17) double-mutant female mice alongside their *Nlgn3*^+/−^ (*n *=* *20), *Cyfip1*^+/−^ (*n = *10), and WT (*n = *16) littermates. The *Nlgn3*^+/−^ mice as well as the *Nlgn3*^+/−^*Cyfip1*^+/−^ mice were no different to their WT littermates in their exploratory behavior in the open field. However, the *Nlgn3*^+/−^ females (day 2: mean = 6129.85 cm, SE = 301.30 cm) and *Nlgn3*^+/−^*Cyfip1*^+/−^ females (day 2: mean = 5059.01 cm, SE = 274.50 cm) were hyperactive in relation to their *Cyfip1*^+/−^ littermates (day 2: mean = 4550.05 cm, SE = 309.99 cm; one-way ANOVA, main effect of genotype: *F*_(3,59)_ = 4.64, *p* < 0.001; Tukey’s HSD: *t*_(1,59)_ = 3.34, *p *<* *0.01, and *t*_(1,59)_ = 2.66, *p *<* *0.05, respectively; Fig. 1*C*). This finding was corroborated when the exploratory behavior across the two days of testing was considered (mixed-model ANOVA, main effect of genotype: *F*_(3,59)_ = 4.51, *p* < 0.001; Tukey’s HSD *Nlgn3*^+/−^ vs *Cyfip1*^+/−^: *t*_(1,59)_ = 3.423, *p *<* *0.01). All of the female mice showed a significant decrease in the distance traveled between the first and second day of testing, characteristic of habituation (WT: *t*_(1,59)_ = 5.61, *p* < 0.001; *Nlgn3*^+/−^: *t*_(1,59)_ = 4.42, *p *<* *0.01; *Cyfip1*^+/−^: *t*_(1,59)_ = 3.83, *p *<* *0.01; *Nlgn3*^+/−^*Cyfip1*^+/−^: *t*_(1,59)_ = 5.30, *p *<* *0.001; Fig. 1*D*). The subtly altered behavior in the female mice cannot be attributed to differences in anxiety levels, as the time spent in the center of the open field was comparable between the different groups (Extended Data Fig. 1-1*B*). Finally, to investigate whether there is a sex difference in the exploratory behavior of these mice, we directly compared the WT and *Cyfip1*^+/−^ males and females; however, we found no significant differences in the distance traveled or the time spent in the center of the open field between males and females (Extended Data Fig. 1-1*C*,*D*). There were no significant differences between *Nlgn3^y/−^* males and *Nlgn3*^+/−^ females neither in their exploratory behavior in the open field (Extended Data Fig. 1-1*E*,*F*).

While *Nlgn3^y/−^Cyfip1*^+/−^ males phenocopied *Nlgn3^y/−^* males in their hyperactivity, the *Nlgn3*^+/−^*Cyfip1*^+/−^ females were no different from their WT littermates. There was no evidence, however, that reducing the levels of *Cyfip1* affected the level of activity in the males, suggesting that only *Nlgn3* influences this behavior.

### Reduction of *Cyfip1* level restores motor learning in *Nlgn3^y/−^Cyfip1*^+/−^ male mice

The ability to learn new motor routines was evaluated by training mice to stay on an accelerating rotating rod, with multiple trials within a day (Extended Data Fig. 2-1) and over 3 consecutive days (Fig. 2). There was no overall effect of genotype on the latency to fall off the rotarod, averaged across days and trials. However, the performance of mice across the days of testing depended on their genotype, indicating that there might be differences in their learning curves (nonparametric mixed-model ANOVA, genotype × day interaction: *F*_(3,1577)_ = 4.61, *p *< 0.01; Extended Data Fig. 2-1*A*). In line with this observation, an increased ability to stay on the rod across multiple days was observed in WT male mice (*n *=* *17; day 1 vs day 2 simple effect: *t*_(1,16)_ = 2.12, *p *<* *0.05, day 1 vs day 3 simple effect: *t*_(1,16)_ = 6.28, *p *<* *0.001; day 2 vs day 3 simple effect: *t*_(1,16)_ = 2.86, *p *<* *0.05), in the *Nlgn3^y/−^* male mice (*n *=* *16; day 1 vs day 2 simple effect: *t*_(1,15)_ = 6.16, *p *< 0.001; day 1 vs day 3 simple effect: *t*_(1,15)_ = 9.14, *p *<* *0.001; day 2 vs day 3 simple effect: *t*_(1,15)_ = 3.02, *p *<* *0.01), and in the *Nlgn3^y/−^Cyfip1*^+/−^ male mice (*n *=* *12; day 1 vs day 2 simple effect: *t*_(1,12)_ = 5.05, *p *<* *0.01; day 1 vs day 3 simple effect: *t*_(1,12)_ = 4.69, *p *<* *0.01; Fig. 2*A*). As previously reported, the *Cyfip1*^+/−^ mice (*n *=* *12) did not improve in their ability to stay on the rod across the days of training, suggesting that they are unable to learn new motor routines within this protocol (Bachmann et al., 2019). These results indicate that the deficit in motor learning seen in *Cyfip1*^+/−^ mice is restored by deleting *Nlgn3* in *Nlgn3^y/−^Cyfip1*^+/−^ double-mutant mice.

Next, we investigated whether a sex difference was present in the ability of these mice to acquire knowledge of new motor routines. To verify this possibility, we included *Nlgn3*^+/−^*Cyfip1*^+/−^ female mice and their littermates in the rotarod training. The genotype did not affect the training across the 3 days, suggesting that the learning curves for all of the females were comparable (Extended Data Fig. 2-1*B*). In line with this observation, an increase in time spent on the rod was observed for WT mice (*n = *13, day 1 vs day 2: *t*_(1,13)_ = 5.09, *p *<* *0.01; day 1 vs day 3: *t*_(1,13)_ = 5.85, *p *<* *0.01), *Nlgn3*^+/−^ mice (*n *=* *13; day 1 vs day 2: *t*_(1,13)_ = 6.15, *p *<* *0.01; day 1 vs day 3: *t*_(1,13)_ = 8.06, *p *<* *0.001; day 2 vs day 3: *t*_(1,13)_ = 4.23, *p *<* *0.05), *Cyfip1*^+/−^ mice (*n = *14; day 1 vs day 3: *t*_(1,13)_ = 5.95, *p *<* *0.01), and *Nlgn3*^+/−^*Cyfip1*^+/−^ mice (*n *=* *14; day 1 vs day 2: *t*_(1,14)_ = 7.34, *p *<* *0.001; day 1 vs day 3: *t*_(1,14)_ = 7.41, *p *<* *0.001; Fig. 2*B*). To determine whether there was a sex difference in the motor learning, we have compared the WT and *Cyfip1*^+/−^ males and females directly. On average, females performed better than the males (nonparametric mixed-model ANOVA, main effect of sex: *F*_(1,52)_ = 4.88, *p *<* *0.05; Extended Data Fig. 2-1*C*). Therefore, unlike males, all female mice showed evidence of learning across days. We also compared the *Nlgn3* mutant mice (*Nlgn3*^+/−^ females and *Nlgn3^y/−^* males) alongside their WT littermates; however, there were no differences in motor learning between the sexes (Extended Data Fig. 2-1*D*).

In male mice, *Nlgn3* and *Cyfip1* act in opposition to control the motor learning on the rotarod. While reducing the level of *Cyfip1* in the males results in impairment in the ability to acquire new motor routines, additional deletion of *Nlgn3* leads to restoration of the behavior. Additionally, sex plays a role in the control of motor learning, where females heterozygous for *Cyfip1* show no deficit.

### Lack of *Nlgn3* and *Cyfip1* haploinsufficiency have little effect on social behavior

**Figure 3.**
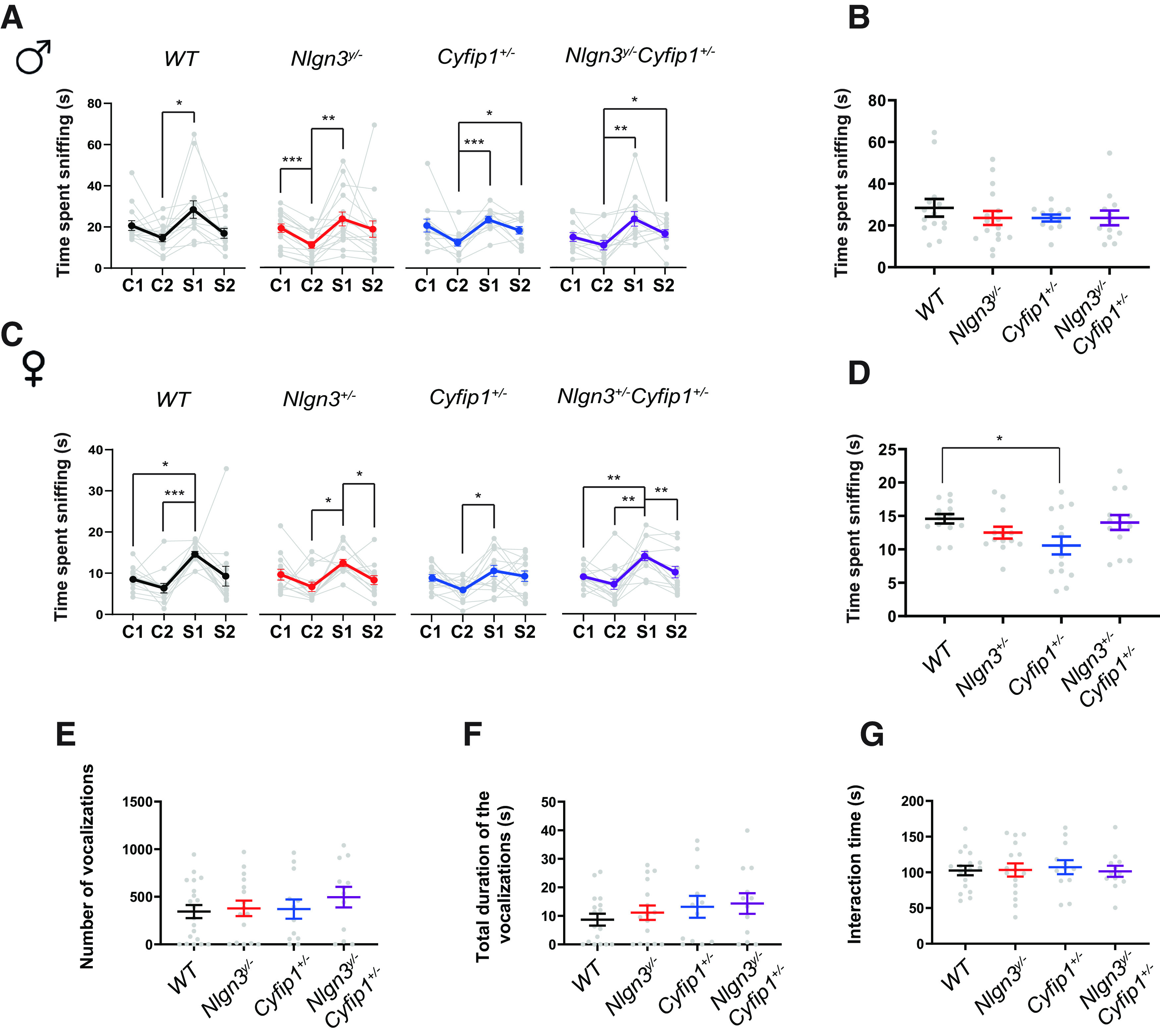
***A***, Time spent sniffing the olfactory cue by the *Nlgn3^y/−^Cyfip1*^+/−^ male mice and their littermates. ***B***, Time spent sniffing during the first presentation of social olfactory cue depending on the genotype by male mice. ***C***, Time spent sniffing the olfactory cue by *Nlgn3^+/−^Cyfip1*^+/−^ female mice and their littermates. ***D***, Time spent sniffing during the first presentation of social olfactory cue depending on the genotype by female mice. ***E***, Number of ultrasonic vocalisations emitted in response to a female in oestrus by the male mice. ***F***, Duration of the ultrasonic vocalisations emitted by male mice. ***G***, Time spent in the interaction with a female by male mice. *p <* *0.05, **p <* *0.01, ***p <* *0.001.

The social behavior of double-mutant mice and their littermates was evaluated by investigating their interest in social odors and, for the males only, their courtship behavior and direct social interaction with a female in estrus. The ability to discriminate between the control and social odor was observed in WT mice (*n *=* *14; C2: mean = 14.61 s, SE = 1.79 s; S1: mean = 28.46 s; SE = 4.23; simple effect: *t*_(1,14)_ = 4.60, *p *<* *0.05), *Nlgn3^y/−^* mice (*n *=* *16; C2: mean = 14.61 s, SE = 1.79 s; S1: mean = 28.46 s, SE = 4.23 s; simple effect: *t*_(1,16)_ = 5.91, *p *<* *0.01), *Cyfip1*^+/−^ mice (*n *=* *12; C2: mean = 12.43 s, SE = 1.72 s; S1: mean = 23.57 s, SE = 1.65 s; simple effect: *t*_(1,12)_ = 8.74, *p *<* *0.001), and *Nlgn3^y/−^Cyfip1*^+/−^ mice (*n *=* *12; C2: mean = 10.88 s, SE = 2.29 s; S1: mean = 23.62 s, SE = 3.54 s; simple effect: *t*_(1,12)_ = 4.40, *p *<* *0.05; Fig. 3*A*). However, habituation to the social odor was observed only in in the *Cyfip1*^+/−^ mice (S1: mean = 23.57 s, SE = 1.65 s; S2: mean = 18.40 s, SE = 1.84 s; simple effect: *t*_(1,12)_ = 4.70, *p *<* *0.05), and the *Nlgn3^y/−^Cyfip1*^+/−^ mice (S1: mean = 23.62 s, SE = 3.54 s; S2: mean = 16.53 s, SE = 1.858 s; simple effect: *t*_(1,12)_ = 4.71, *p *<* *0.05). The level of interest in the social odor was also the same between the different genotypes (Fig. 3*B*).

The genotype of the female mice had no impact on their ability to discriminate between control and social odors. An increase in the time spent with the cue was observed between the control cue and the first presentation of the social odor in WT mice (*n *=* *13; C2: mean = 6.37 s, SE = 1.15 s, S1: mean = 14.57 s, SE = 0.70 s; simple effect: *t*_(1,13)_ = 10.80, *p *<* *0.001), *Nlgn3*^+/−^ mice (*n *=* *13; C2: mean = 6.71 s, SE = 1.13 s; S1: mean = 12.50 s, SE = 0.89 s; simple effect: *t*_(1,13)_ = 5.50, *p *<* *0.01), *Cyfip1*^+/−^ mice (*n *=* *14; C2: mean = 5.96 s, SE = 0.69 s; S1: mean = 10.57 s, SE = 1.33 s; simple effect: *t*_(1,14)_ = 4.82, *p *<* *0.05), and *Nlgn3*^+/−^*Cyfip1*^+/−^ mice (*n *=* *14; C2: mean = 7.18 s, SE = 1.23 s; S1: mean = 14.01 s, SE = 1.12 s; simple effect: *t*_(1,14)_ = 5.44, *p *<* *0.01; Fig. 3*C*). In the female mice, habituation was only observed in *Nlgn3*^+/−^ mice (S1: mean = 12.50 s, SE = 0.89 s; S2: mean = 8.34 s, SE = 1.08 s; simple effect: *t*_(1,13)_ = 4.80, *p *<* *0.05) and *Nlgn3*^+/−^*Cyfip1*^+/−^ mice (S1: mean = 14.01 s, SE = 1.12 s; S2: mean = 10.10 s, SE = 1.40 s; simple effect: *t*_(1,14)_ = 6.39, *p *<* *0.01). The interest in the social odor was reduced in the *Cyfip1*^+/−^ females compared with the WT females (one-way ANOVA: main effect of genotype: *F*_(3,50)_ = 2.93, *p *<* *0.05; Tukey’s HSD: *t*_(1,50)_ = −2.68, *p *<* *0.05; Fig. 3*D*).

The courtship behavior of the males exposed to a female in estrus was equivalent in all the mutants (*Nlgn3^y/−^*, *n *=* *16; *Cyfip1*^+/−^, *n *=* *12; *Nlgn3*^+/−^*Cyfip1*^+/−^, *n *=* *12) and their WT littermates (*n *=* *17). Specifically, the number and the duration of ultrasonic vocalizations emitted in response to the female as well as the time spent in direct interaction with the female was not different between the WT littermates and the other males (Fig. 3*E–G*). Deficits in courtship behavior in *Nlgn3^y/−^* males were reported multiple times (Radyushkin et al., 2009; Fischer and Hammerschmidt, 2011; Kalbassi et al., 2017). In contrast to these findings, there is no evidence for a deficit in courtship vocalization in the *Nlgn3^y/−^* males here. However, it is important to note that while the number and duration of vocalization in the *Nlgn3^y/−^* males are comparable here to the previous literature, the level of vocalization in the WT littermates is lower than expected. The reduced level of vocalization during courtship in the WT males might be a result of being housed with mutant animals, similar to a previously reported effect of the social environment (Kalbassi et al., 2017).

In opposition to previous literature, neither *Nlgn3* nor *Cyfip1* had a substantial impact on social behavior in male mice. This could be because of the effect of the social environment modulating the behavior of the WT controls as well as potentially the mutant animals.

### *Nlgn3 and Cyfip1* collectively impact on dendritic spine density in the motor cortex

**Figure 4.**
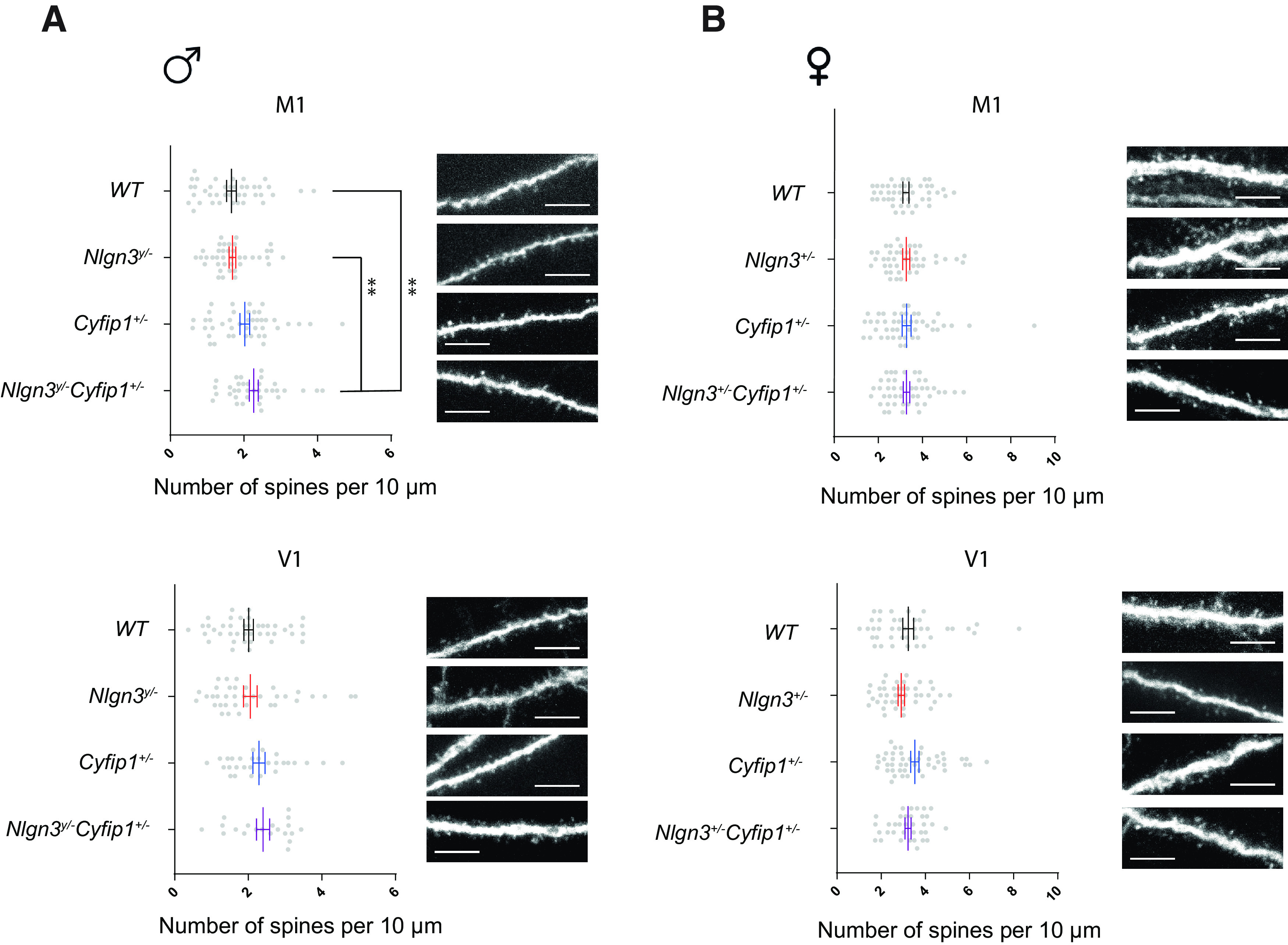
The effect of *Nlgn3* deletion and *Cyfip1* haploinsufficiency on spine density in the cortex. ***A***, The dendritic spine density in the M1 area of motor cortex and the V1 area of visual cortex in *Nlgn3^y/-^Cyfip1*^+/−^ males and their littermates. ***B***, The dendritic spine density in the M1 area of motor cortex and the V1 area of visual cortex in *Nlgn3*^+/−^*Cyfip1*^+/−^ females and their littermates. Scale bars, 10 μm. *p <* *0.05, **p <* *0.01, ***p <* *0.001. The sex comparison is available in Extended Data Figure 4-1.

Altered dendritic spine regulation is another phenotype associated with ASDs (Phillips and Pozzo-Miller, 2015). We investigated the impact of deleting *Nlgn3* and *Cyfip1* haploinsufficiency on dendritic spine density. For this purpose, we obtained *Nlgn3^y/−^Cyfip1*^+/−^ double-mutant mice as well as their *Nlgn3^y/−^*, *Cyfip1*^+/−^, and WT littermates, where the EGFP transgene was expressed in a subset of neurons, allowing the visualization of dendritic spines. Changes in dendritic spine density, as well as turnover, have previously been reported in the motor cortex of *Cyfip1*^+/−^ male mice (Bachmann et al., 2019); thus, this brain region was included in the analysis. No changes in the visual cortex were reported in these mice, hence we included this region as a control. In the motor cortex, *Nlgn3^y/−^Cyfip1*^+/−^ males had a significantly greater density of spines per 10 μm of dendrite (mean = 2.27, SE = 0.12) than *Nlgn3^y/−^* males (mean = 1.69, SE = 0.09; Kruskal–Wallis test: χ^2^_(3153)_ = 15.64, *p < *0.01, *z*_(1153)_ = −3.27, *p *<* *0.01) and WT males (mean = 1.66, SD* *=* *0.13; *z*_(1153)_ = −3.41, *p *<* *0.01; Fig. 4*A*). No significant differences in spine density were observed in the visual cortex, emphasizing the regional specificity of the effect. In contrast to the males, no significant differences in spine density in the motor or visual cortex were present in the females (Fig. 4*B*). To confirm the sex difference, we compared the numbers of dendritic spines in the male and female mice directly. The WT and *Cyfip1*^+/−^ females had on average more dendritic spines in the cortex than the WT and *Cyfip1*^+/−^ males (Scheirer–Ray–Hare test, main effect of sex: *H*_(1326)_ = 98.97, *p *<* *0.001; Extended Data Fig. 4-1). The selectively reduced numbers of dendritic spines in male mice raised the possibility that the social environment might impact on this phenotype, whereby being raised with their mutant littermates, WT males showed lower than expected density of dendritic spines.

10.1523/ENEURO.0124-20.2020.f4-1 Figure 4-1 The effect of sex on spine density in the cortex. The dendritic spine density in the cortex in WT and *Cyfip1*^+/–^ male and female mice. Download Figure 4-1, EPS file.

### Lack of *Nlgn3* and *Cyfip1* haploinsufficiency shapes the transcriptome profiles in male mice

We investigated the effect of lack of *Nlgn3* and *Cyfip1* haploinsufficiency as well as the combined effect of both on RNA expression. Previous results raised the possibility that the social environment might affect the behavior and the spine density of the WT mice, potentially impacting on the interpretation of these findings (Kalbassi et al., 2017). To investigate the possibility that the social environment might impact on the transcriptome, we included a control group of WT males that were housed only with their WT littermates [single genotype housing (SGH)] in addition to WT males that were housed with mutant animals [mixed genotype housing (MGH)]. We performed RNA sequencing of the brain tissue, specifically the hippocampus, in *Nlgn3^y/−^Cyfip1*^+/−^, *Nlgn3^y/−^*, and *Cyfip1*^+/−^ males as well as in SGH and MGH WT mice. We have selected the hippocampus as a region of interest, as it is well recognized for being sensitive to environmental changes. Moreover, hippocampal functions have been associated with the emergence of individuality in genetically identical wild-type mice (Freund et al., 2013; Kempermann, 2019). Thus, we hypothesized that differences in the social environment of wild-type mice could affect the RNA profile in the hippocampus.

**Figure 5.**
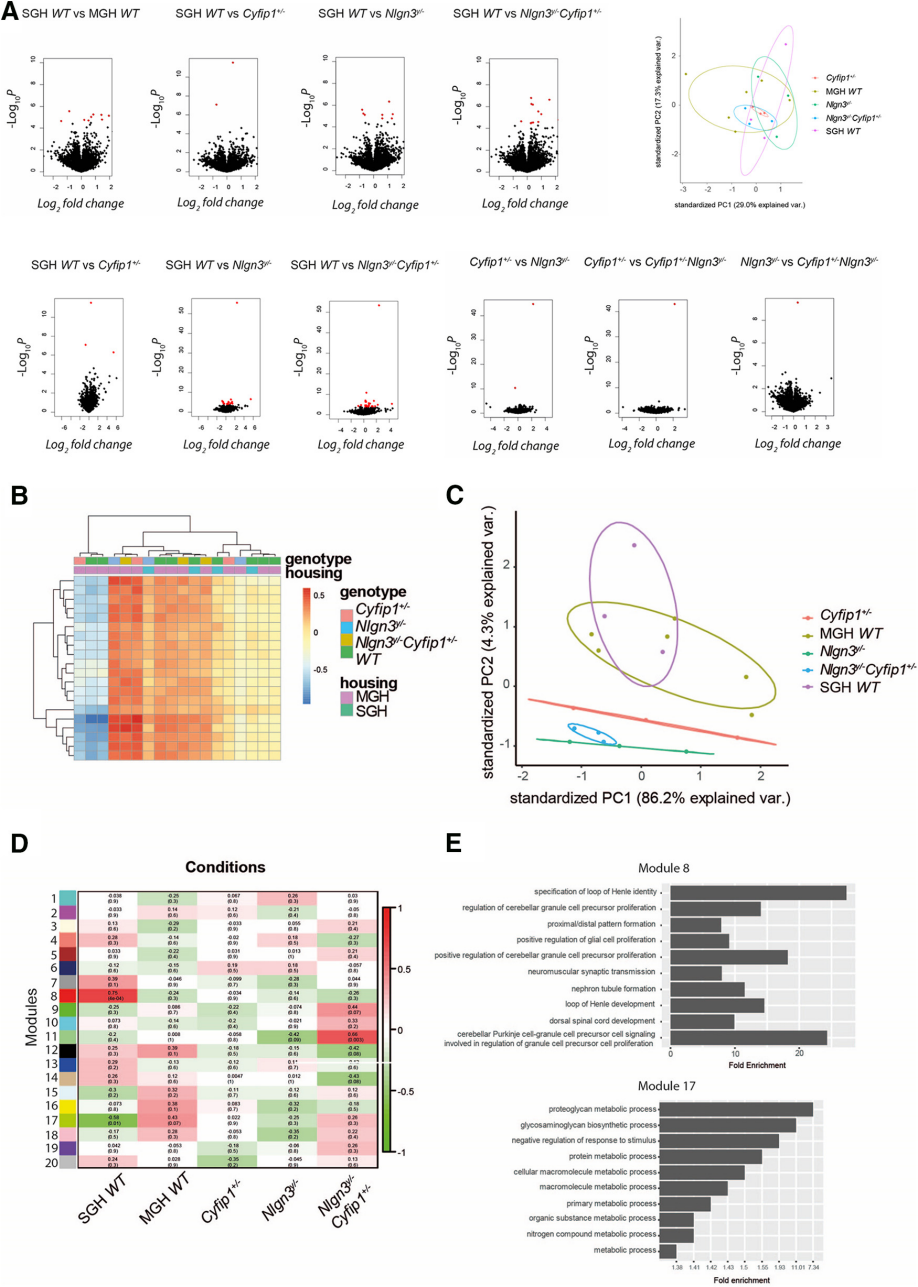
Nlgn3 and Cyfip1 mutations affect the transcriptome profile of male mice. ***A***, Differentially expressed genes between the SGH WT, MGH WT*, Cyfip1*^+/−^, *Nlgn3^y/−^*, and *Nlgn3^y/−^Cyfip1*^+/−^ male mice. ***B***, Hierarchical clustering of differentially expressed genes in the different housing and genotype conditions. ***C***, Position of the individual samples from different housing and genotype conditions in the principal component space based on the 100 genes with the greatest fold change. ***D***, WGCNA modules correlation with the different housing and genotype combinations. ***E***, GO pathways for modules correlated with SGH WT condition.

Initially, we compared the number of differentially expressed genes in the hippocampi of males from different conditions. First, we compared the mutant mice to their WT littermates (MGH WT). There were very few differentially expressed genes between the mutant mice and the MGH WT controls (*Cyfip1*^+/−^ vs MGH WT: two upregulated, zero downregulated; *Nlgn3^y/−^Cyfip1*^+/−^ vs MGH WT: three upregulated, zero downregulated; *Nlgn3^y/−^* vs MGH WT: four upregulated, 1 downregulated; Fig. 5*A*). The differences between the MGH and SGH WT males were, however, more substantial, with 15 upregulated and 3 downregulated genes. This difference suggests that housing conditions have the capacity to shape transcription profiles. Thus, next, we compared the mutant animals to the SGH WT controls. The differences between the SGH WT animals and the *Cyfip1*^+/−^ animals were still small (SGH WT vs *Cyfip1*^+/−^: 2 upregulated, 1 downregulated), while the differences between the WT males and *Nlgn3^y/−^* animals were more substantial (SGH WT vs *Nlgn3^y/^*: 12 upregulated, 5 downregulated). There were also some differences between SGH WT controls and the *Nlgn3^y/−^Cyfip1*^+/−^ double mutants (SGH WT and *Nlgn3^y/−^Cyfip1*^+/−^: 21 upregulated, 2 downregulated), suggesting that while *Cyfip1* haploinsufficiency has little impact on the transcription profile, the lack of *Nlgn3* has a role in shaping it. Despite the presence of differentially expressed genes, a clear separation based on housing or the genotype of the mice was not evident in the hierarchical clustering (Fig. 5*B*). However, a degree of separation was present following principal component analysis, with SGH WT and MGH WT samples in particular occupying nonoverlapping space (Fig. 5*C*).

In the course of the analysis of a differentially expressed gene, a wealth of information about the coregulation between different genes is lost because of using an arbitrary *p* value level. To investigate the similarities between the transcription profiles in more depth, we used a weighted gene correlation network analysis (WGCNA). We constructed a coexpression network that contained 20 modules. Of these, the lack of *Nlgn3* was significantly associated with module 10 [*R*^2^ = −0.42, false discovery rate (FDR) = 0.09], while the reduced level of *Cyfip1* was not linked to any of the modules. However, when the two mutations were combined in the *Nlgn3^y/−^Cyfip1*^+/−^ mutant mice, module 11 (*R*^2^ = 0.66, FDR = 0.003), module 13 (*R*^2^ = −0.42, FDR = 0.08), and module 15 (*R*^2^ = −0.43, FDR = 0.08) were all significantly associated with this trait, suggesting an additive effect of the two mutations (Fig. 5*D*). When the housing condition was considered, module 8 was found to be upregulated in the SGH animals compared with the MGH animals (*R*^2^ = 0.75, FDR < 0.01), while module 17 was downregulated (*R*^2^ = −0.58, FDR = 0.01; Fig. 5*E*). The differential expression of the modules of genes depending on housing and genotype of the males suggests that both the social environment and the presence of *Nlgn3* and *Cyfip1* impact on the transcription profile. The genes in module 8 were associated with development, in particular with the development of the loop of Henle as well as synaptic transmission and cerebellar cell proliferation and signaling, while the genes in module 17 were associated with metabolic processes (Fig. 5*E*).

While there were some differences between the transcription profile arising from mice with different genetic mutations, the effect of housing was more pronounced.

### Social environment impacts on transcriptome profiles in male mice

**Figure 6.**
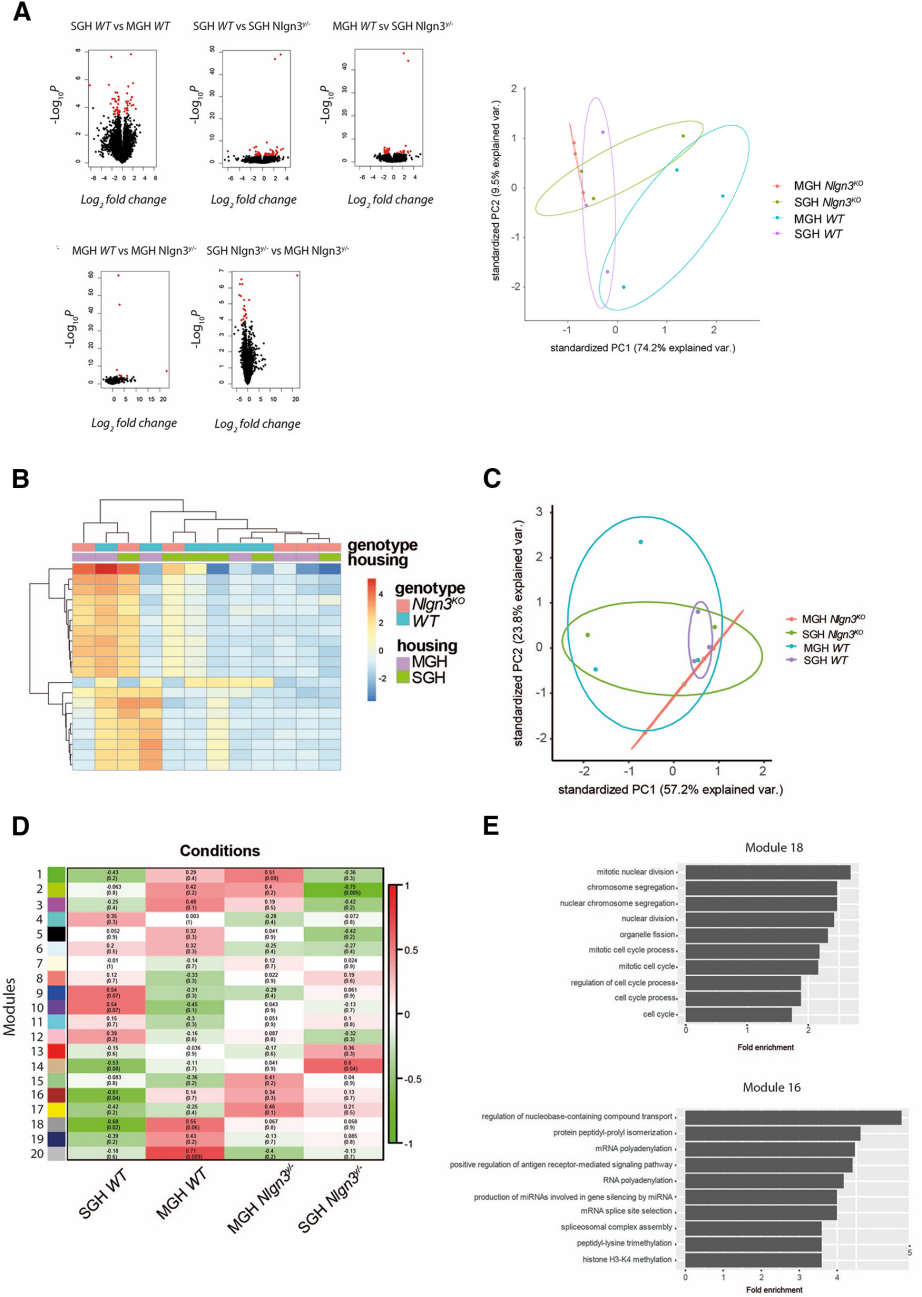
Social environment affects the transcriptome profile of WT and *Nlgn3^y/−^* mice. ***A***, Differentially expressed genes in the SGH WT, MGH WT, SGH *Nlgn3^y/−^*, and MGH *Nlgn3^y/−^.* ***B***, Hierarchical clustering of differentially expressed genes in the different housing and genotype conditions. ***C***, Position of the individual samples from different housing and genotype conditions in the principal component space based on the 100 genes with the greatest fold change. ***D***, WGCNA module correlation with the different housing and genotype combinations. ***E***, GO pathways for modules correlated with SGH WT condition.

We have found that WT males housed with their WT littermates have a distinctly different transcription profile from WT animals housed with mutant animals as well as the mutant animals themselves. However, the previous experiment used a cohort of WT animals from a different breeding line than the mutant animals, potentially artificially increasing the differences between the SGH and MGH WT mice. Thus, we attempted to replicate the effect of the social environment on the transcriptome in SGH and MGH WT males that came from the same breeding line and thus had the same mothers as each other as well as mutant males. Additionally, we included the single and mixed genotype housing condition for the *Nlgn3^y/−^* males (SGH *Nlgn3^y/−^* and MGH *Nlgn3^y/−^*) to investigate the potential impact of the social environment on the mutant mice. As previously, there was a fair number of genes that were differentially expressed between the MGH and SGH WT males (23 upregulated, 34 downregulated), suggesting that housing conditions shaped the transcription profiles in this group (Fig. 6*A*). The effect of housing was also evident to a smaller extent in the *Nlgn3^y/−^* males, where there were 2 upregulated and 18 downregulated genes between SGH *Nlgn3^y/−^* and MGH *Nlgn3^y/−^* males. While between the SGH WT and SGH *Nlgn3^y/−^* males that were never housed with mice of the same genotype, there were many differentially expressed genes (39 upregulated and 18 downregulated), and there were very few differences between the MGH WT and MGH *Nlgn3^y/−^* mice that were housed together (8 upregulates, 0 downregulated). These findings suggest that the social environment impacts the transcriptome profile not only in the WT littermates but also in the mutant *Nlgn3^y/−^* mice. Despite the presence of a number of differentially expressed genes, a clear separation based on housing was not evident in the hierarchical clustering (Fig. 6*B*). A degree of separation was present following principal component analysis with SGH WT and MGH WT samples in particular occupying nonoverlapping space (Fig. 6*C*). WGCNA resulted in a network that contained 20 modules. In SGH WT animals, modules 9 and 10 were significantly upregulated (*R*^2^ = 0.5, FDR = 0.09), and modules 14 (*R*^2^ = −0.53, FDR = 0.08), 16 (*R*^2^ = −0.61, FDR = 0.04), and 18 (*R*^2^ = 0.68, FDR = 0.02) were significantly downregulated (Fig. 6*D*). In SGH *Nlgn3^y/−^* males, module 14 (*R*^2^ = 0.60, FDR = 0.04) was significantly upregulated and module 2 was significantly downregulated (*R*^2^ = −0.75, FDR = 0.005; Fig. 6*D*). Meanwhile in the MGH WT animals, modules 18 and 20 were significantly upregulated (*R*^2^ = 0.55, FDR = 0.06; and *R*^2^ = 0.71, FDR = 0.009, respectively), and in the MGH *Nlgn3^y/−^* animals, module 1 was upregulated (*R*^2^ = 0.51, FDR = 0.09). The differential expression of the modules of genes depending on housing suggests that the social environment impacted on the transcription profile. The two modules most strongly associated with SGH WT males were module 18-containing genes responsible for cell cycle processes and chromatin regulation and module 16-containing genes associated with RNA regulation. As evident from the different GO terms associated with the SGH WT males in this cohort and the previous cohort, some of the genes in the relevant modules were different depending on the cohort.

Here we show that lack of *Nlgn3* has an impact on the RNA expression in the hippocampus, while *Cyfip1* haploinsufficiency does not. Additionally, we extend the previous finding that the presence of *Nlgn3^y/−^* littermates influences the social behavior of their WT littermates (Kalbassi et al., 2017) to potentially include an effect on the transcription profile. Together, these findings suggest a complex genetic–environment interaction that shapes the RNA expression.

## Discussion

In the first attempt of this kind, we investigated the combined effect of genetic and environmental factors on the phenotypes associated with ASDs. We uncovered that the *Nlgn3/Cyfip1* pathway plays a role in mouse behavior, dendritic spine density, and RNA expression. We found that sex can be a protective factor, where the females carrying the risk allele do not show the same deficits as the males. The behavior and transcriptome of WT mice is further influenced by the social group they originate from, suggesting that the social environment is an important factor modulating the phenotypes in mouse models.

As in previous literature, we found that male mice lacking *Nlgn3* engage in more exploratory behavior in the open field (Radyushkin et al., 2009; Kalbassi et al., 2017) and that male mice with a reduced level of *Cyfip1* expression are unable to learn motor routines (Bachmann et al., 2019). We have extended these findings to show that, unexpectedly, the accumulation of mutations can lead to a correction of the motor learning deficit. The interaction between Neuroligin3 and CYFIP1 at the synapse has been previously reported (Bachmann et al., 2019). The current findings support the idea that the consequences of this protein interaction extend beyond the molecular events, resulting in a change in the behavior of the animal. As the genetic architecture of ASDs is complex and far from being entirely understood, it is increasingly important to consider the interaction between the different proteins involved and how they shape the phenotypes seen in ASDs.

**Figure 7.**
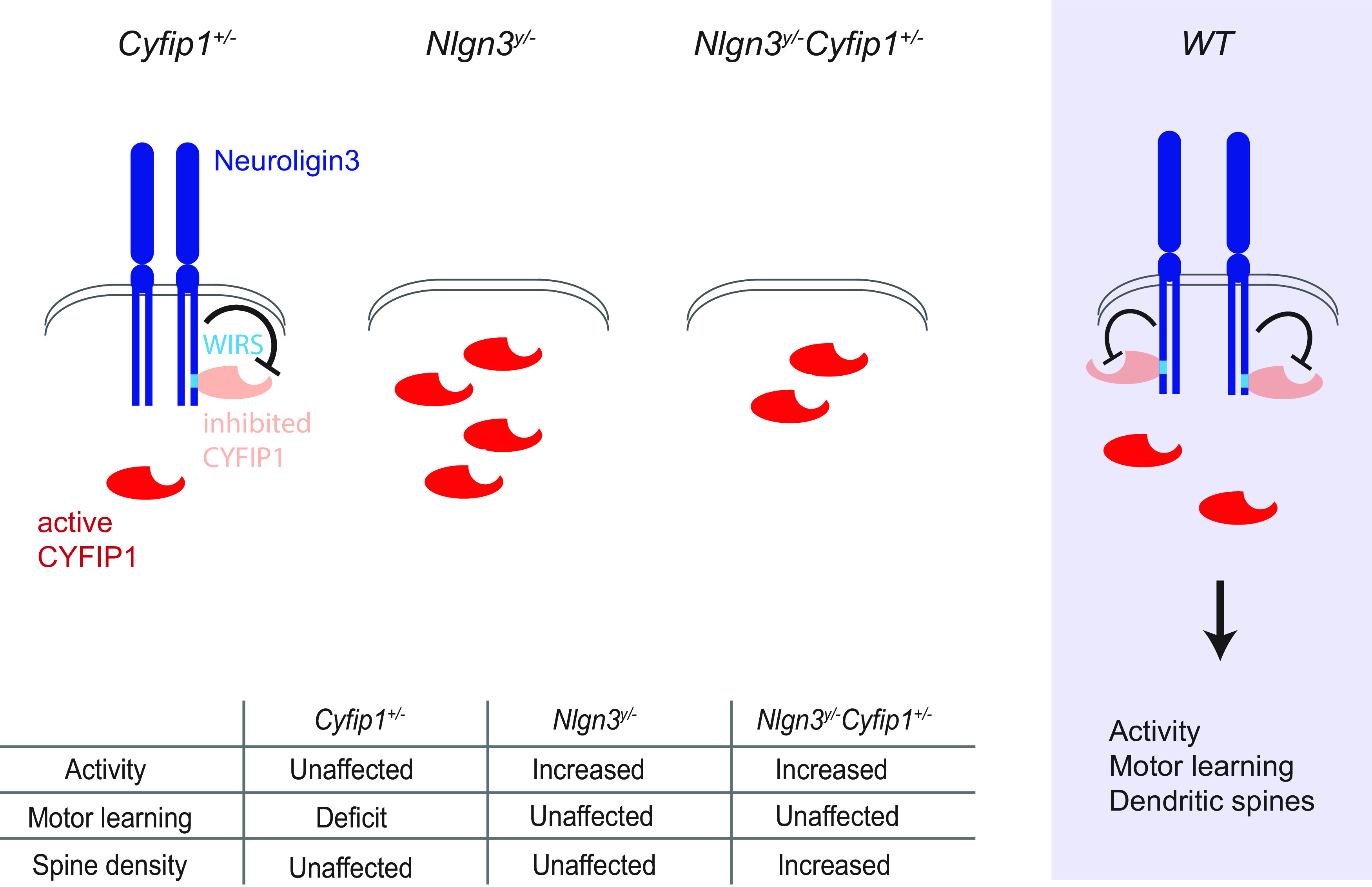
Neuroligin3 inhibits CYFIP1 in the population of neurons important for motor learning. In *Cyfip1*^+/−^ males, there is a deficit in motor learning, potentially because Neuroligin3 inhibits the already reduced pool of available CYFIP1. In the *Nlgn3^y/−^* males, the lack of inhibition of CYFIP1 results in motor learning, accompanied by hyperactivity. In the double-mutant males, the remaining CYFIP1 is not inhibited by Neuroligin3, resulting in the restoration of motor learning that is still, however, accompanied by hyperactivity and an increase in dendritic spine density. Thus, in the WT Neuroligin3 is likely to inhibit CYFIP1, regulating motor learning activity levels as well as dendritic spine density in the cortex.

The behavioral results suggest that the nature of the functional relationship between Neuroligin3 and CYFIP1 is inhibitory. In the males with *Cyfip1* haploinsufficiency, the level of CYFIP1 was found to be reduced in several brain areas (Bachmann et al., 2019). We found that this decrease resulted in a deficit in motor learning that was accompanied by unaltered levels of activity and dendritic spine density in the cortex (Fig. 7). This suggests that in the males heterozygous for *Cyfip1*, the Neuroligin3 might be binding some of the available CYFIP1 in the cellular population important for motor learning, preventing it from performing its physiological function and resulting in a deficit. In the males with *Nlgn3* deletion, on the other hand, physiological levels of CYFIP1 result in the mice being able to learn the motor routines. However, the lack of Neuroligin3 might result in increased levels of available CYFIP1, potentially leading to the increased activity levels seen in this model. The alternative explanation is that the increase in activity results from another pathway regulated by Neuroligin3. In the double-mutant males, which lack *Nlgn3* and are heterozygous for *Cyfip1*, the increase in activity and dendritic spine density is accompanied by normalized motor learning. In this model, the level of CYFIP1 is likely reduced, but there is no Neuroligin3 to inhibit the remaining CYFIP1. As a result, there is enough CYFIP1 available to restore motor learning. Meanwhile, the lack of Neuroligin3 also results in an increased level of activity. Interestingly, only the two mutations together result in an increase of dendritic spine density. These results suggest that in the WT animals, Neuroligin3 inhibits the portion of available CYFIP1, regulating motor learning as well as potentially activity levels and dendritic spine density in the cortex.

We showed that while male mice with reduced levels of *Cyfip1* have a clear phenotype, females with the same genotype are capable of learning motor routines as usual. Additionally, our data suggest that there might be a reduction in the number of dendritic spines in the males compared with the females in the cortex. While this finding needs to be confirmed in a cohort of wild-type animals, accounting for the social dominance status, it implies that only males are affected by carrying a risk allele. While a sex difference in ASDs is frequently reported, with the affected male to female ratio being 4:1, remarkably little is known about the source of this sex difference (Halladay et al., 2015). The female protective effect is one theory aiming to explain this discrepancy, whereby females require more substantial disruption of the genetic network and the associated biological pathways to show ASD symptoms (Ferri et al., 2018). Some pathways relevant for ASDs might be redundant in females, as a number of genes associated with ASDs are X linked. While *Cyfip1* is present on chromosome 7, it interacts with two X-linked genes: *Nlgn3* and *Fmr1. Cyfip1* being part of the X-linked pathway results in unimpaired females likely because they carry two rather than one allele of the interacting genes.

In opposition to the previous report, *Cyfip1* haploinsufficiency did not affect the spine density in the motor cortex of the male mice (Bagni and Greenough, 2005; Pathania et al., 2014; Bachmann et al., 2019). While in the previous report *Cyfip1* haploinsufficiency resulted in a decrease in the dendritic density in the motor cortex, here the double-mutant mice showed an increase in dendritic spine density. The reliability of this finding is undermined by the low numbers of dendritic spines in the male mice of 1–5 spines per 10 μm, while the numerous previous studies reported a density of 5–15 spines per 10 μm both in the males heterozygous for *Cyfip1* and in WT males (De Rubeis et al., 2014; Pathania et al., 2014; Abekhoukh et al., 2017). This was true only for the male mice, while the sample from the female mice showed a range of 2–10 spines per 10 μm, which is in line with the previous reports. The low number of dendritic spines is unlikely to be because of the technical difficulties, as the male and female mice used in the experiment were littermates, came from the same line, and were analyzed in parallel. Therefore, it is unlikely that the low numbers in the males but not in the females are a technical artifact. The alternative explanation is that there is another factor selectively impacting the spine density in the WT males. One such factor could be the social environment. The effect of the social environment in the mouse models of ASDs has not been extensively studied. However, there were some reports of mutant animals, including males lacking *Nlgn3*, impacting on the behavior of the WT littermates (Yang et al., 2011; Kalbassi et al., 2017). The biological processes underlying mouse behavior are complex; however, there is a possibility that the density of dendritic spines might be correlated with behavior. Therefore, the low number of dendritic spines seen in the WT mice housed with the mutant littermates might be the result of the social environment. To confirm that the social housing effect can extend to the dendritic spine density, it would be necessary to extend the analysis to a cohort of WT males that have never been housed with mutant littermates.

In addition to the possible effect of the social environment on behavior and dendritic spine density, social housing also impacts on the transcriptome in the hippocampus. While the effect of the social environment on the gene expression was replicated in two separate cohorts of animals, the identity of the genes associated with different housing conditions varied. In the first cohort, single-genotype housing of the WT animals was associated with synaptic transmission and metabolic processes, and in the second cohort the same trait was associated with cell cycle processes and RNA regulation. The differences might arise from the fact that one of the two cohorts of SGH WT arose from the same breeding line as the corresponding MGH WT animals while the other cohort did not. We found that wild-type individuals could potentially be differentiated based RNA expression, indicating that the different social environments have a distinct impact on wild-type mice. Although this experiment does not draw a direct link between the identity of the RNA profile and the phenotypes, we could postulate that wild-type behavior, shaped by its social environment, can be underlined by the activation of specific molecular pathways that remain to be determined.

Here we showed that not only genetic factors but also sex and social environment play a fundamental role in shaping the phenotypes of different mouse models of ASDs. Of these factors, the most complex to consider is arguably the role of the social environment. Together with a previous published study (Kalbassi et al., 2017), our experiments suggest that the presence of mutant animals influence wild-type animals in that they adopt behavioral, cellular, and molecular phenotypic traits of mutant animals. The opposite effect, from the mutant to the wild type, seems to occur as well. The resulting effect might be that when the animals with different levels of sociability are placed in a common social context, a homogenization of their behavior, underlined by neuronal morphology and transcriptome profile changes, occurs to set an optimal level of functioning within the social group. This remains a hypothesis that needs to be validated and extended to social groups in general. But if our postulate were to be confirmed, these findings could have important implications regarding experimentation using laboratory animals and, in particular, could lead to re-evaluation of the use of wild-type animals as controls to define phenotypic traits of mouse models.

## Synthesis

Reviewing Editor: Carmen Sandi, Swiss Federal Institute of Technology Decisions are customarily a result of the Reviewing Editor and the peer reviewers coming together and discussing their recommendations until a consensus is reached. When revisions are invited, a fact-based synthesis statement explaining their decision and outlining what is needed to prepare a revision will be listed below. The following reviewer(s) agreed to reveal their identity: Sebastiano Bariselli, Ewelina Knapska.

The manuscript describes the effects of Nlgn3 deletion and Cyfip1 haploinsufficiency, as well as their interaction on exploratory, motor and social behavior, dendritic spine density in the motor and visual cortex and transcriptome profiles. An asset of the study is a comparison of the genetic effects between sexes, as well as testing the effects of housing with other genotypes.

Specifically, the study analyzes behavioral, neuronal and molecular characteristics of two different transgenic models related to autism spectrum disorders (ASDs), including three main facets: i) characterization of behavioral deficits and interaction between Nlg3/Cyfip and motor learning; ii) analyses of spine density in the motor cortex and again investigate the effects of Nlg3/Cyfip KO; iii) transcriptomic analyses of hippocampal cells and its modulation by housing condition.

The two reviewers and review editor consider the results novel and potentially interesting, advancing the field, particularly in the authors’ attempt to assessing the interaction between housing-conditions and genetic background on transcriptome profile. However, we also find that several issues require attention before publication:

General comments:

-As you will see below, we identify a strong need to deal with between-groups statistical analyses (male vs female, etc...) and to avoid drawing conclusions based on under-powered tests. The authors should perform the statistical analysis as suggested below and provide appropriate result interpretation.

-Structural analyses: we are concerned about the low spine density reported in WT mice. As you can see in our comments below, we consider that a deeper characterization of dendritic spine morphology in motor cortex would be important to implement. However, if this comes at a too high cost for your lab at the moment (reasons of confinement or other), you should at least carefully address whether the low-spine density (compared to previous reports) depends on housing-condition, at least in WT animals. If you have the possibility of addressing these issues experimentally, that would be ideal. Alternatively, it will be important that you address the issue very carefully and thoroughly in the Discussion section.

-We also feel that there is a disconnection between different parts of the paper and would like to see a revised version in which all parts are integrated, at least in the discussion. In particular, please make sure you do so to discuss the relationship between motor learning and spine density in hippocampus (early work from Moser) and motor cortex, and how maladaptive spine density can result in the motor impairments observed in your ASD models.

Specific explanation of major points to address:

1. Stats: One of the main results reported in the manuscript is male-female difference in exploratory and motor behavior, however, no direct statistical comparisons are made. Such comparisons are necessary to tell whether the differences exist. Otherwise, the ms. should not present any statement making sex-related comparisons. Please, pay careful attention to this point.

2. In the motor learning results, statistical comparisons between genotypes are missing. In addition, the authors report that WT male mice have a lower spine density in the motor cortex compared to previous reports. The ms. indicates that the lower spine density in the motor cortex reported here for WT male mice may be due to housing conditions. This interpretation implies that the observed increase of dendritic spines in the double-transgenic animals might result from a lower basal level in WT animals. To justify the statement that the reduced density of spines in male mice was caused by social environment, the spine density analysis should be carried out in mice housed with the same and other genotype (as for the transcriptome analysis). The authors should provide more rigorous evidence by analyzing spine density in SGH (single genotype housing) and MGH (multiple genotype housing) WT and transgenic animals. These analyses will clarify the effects of social environment on spine density in motor cortex of ASD animal models.

3. We encourage the authors to explore the existence of a potential link between spine density and motor learning deficits in the transgenic mouse lines. In fact, while the heterozygote elimination of Nlg3 rescues Cyfip+/-, the same mutation appears to further enhance spine density compared to WT or single-transgenic. The authors are encouraged to analyze spine morphology and classify spines as filopodia, mushroom, stubby, etc. This will help to better understand whether there is a persistence of immature spines in the double transgenic, or whether those spines have the morphology of a mature (and functioning) spine.

4. We appreciate the remarkable effort in analyzing the transcriptome of those different ASD mouse models. However, the link of these results with motor learning impairment and spine density is missing. We understand that this is a complex task, but would advise testing the effects of different genotypes and housing conditions on spine density in the hippocampus (eg in CA1 neurons).

5. Results need to be clarified: In the experiments presented in figs 5 and 6, the wild type animals from a different breeding line than the mutant animals and from the same breeding line as the mutant animals were used, respectively. The results section describes which ‘modules’ were up- and down-regulated. In the former experiment the modules are 8 and 17 and, in the latter - 9,10/14,16,18. From the description on the figs, we assume that they are different sets of genes. It is not clearly stated in the manuscript and the meaning of the difference between housing conditions that depend on the breeding line is not discussed.

6. In the discussion, the authors should provide a more thorough explanation on why the interaction between Cyfip and Nlg3 may be important, especially considering the ability of Nlg3y/- to rescue Cyfip+/- motor learning in males and the inability of Cyfip+/- to rescue Nlg3y/- hyperactivity.

Minor points:

7. According to the DMS-5, a deficit in cognitive flexibility is not per sè a symptom that might lead to the diagnosis of a disorder in the autistic spectrum. The two common symptoms of ASDs are social communication deficits and stereotyped behavior. Although stereotypies might be behavioral manifestations of impairments in cognitive flexibility, the latter term refers to a more complex phenotype that includes (but is not restricted to) action repetition, reversal-learning deficits in cue-outcome or action-outcome associations and deficits in adaptations of action strategies. I encourage the authors to use appropriate terms when referring to the ASD symptomatology.

8. The nomenclature of the transgenic lines utilized in this manuscript somewhat confusing. The authors refer to the experimental group of Nlg3 heterozygous KO as Nlg3y/-, Nlg3+/- and Nlg3HET. I invite the authors to use the same nomenclature throughout the text and figures, unless justified. Moreover, the use of the nomenclature Nlg3y/-,Cyfip+/- in reference to the double-transgenic line is confusing, because of the use of commas (,) as punctuation elements in the text. To facilitate a clear reading and understanding of the results, I invite the authors to refer to the double-transgenic line as Nlg3y/-::Cyfip+/-, or other clearer nomenclature of their choice.

9. Please, specify why the RNA sequencing is a technique “less prone to variability”? In what context? Why is it important here?

10. Please, justify why they performed RNAseq of hippocampal tissue. Considering both the effects on behavior and dendritic spine, a more logical choice would have been the analysis of the motor cortex transcriptome.

11. The number of animals should be clearly stated in the results section and/or figures.

2. The age of mice is described as P60 or older - the age range should be given more precisely.

3. Method, Animals - lack of an effect of the transgene on the behavioural outcomes was confirmed - this should be explained.

4. Ultrasonic vocalisation - frequency of the vocalisations should be reported

- Please, refrain the use of “normal behavior” when it does not refer to a sample distribution (normal as in parametric). The authors are encouraged to adopt sentences like: “similar to controls” or “physiological”.

5. Table 1-1 should better explain why in some cases (typically when performing one-way ANOVA) they assumed the normality of sample distributions, while in some other cases they did not and applied the non-parametric Kruskal-Wallis. What do they exactly mean by: “normality assumed based on histogram”?

6. Results, p. 10 - when the comparisons between mutants and wt mice are described, it is said for both Nlgn3 and double mutants: compared to their WT littermates, which suggests two WT groups but from the analysis it seems that there was only one WT group. It should be clarified.

7. Results, p.11 - ‘Unlike the male mice, all of the female mice showed a significant increase in the distance travelled between the first and second day of testing’ is inconsistent with what is shown in Fig 1D.

8. Throughout the manuscript there were many typos and grammar errors. The authors should consider carefully revising and correcting the manuscript.

Extended data: In the extended data sets, the authors included additional analysis of behavioral parameters, including time spent in different zones of the open field and latency to fall from the rotarod. These data-sets are well placed in the extended data as they do not constitute the core of the study, but rather complement the behavioral data reported in the main figures

## Author Response

Dear editor and reviewers,

Thank you very much for your helpful comments on our manuscript. We have revised the manuscript according to your suggestions, please refer to the yellow highlights in the word document. We feel your input have enhanced the article. We continue to believe that this is an important topic in the field, and we hope that the editors and reviewers share our conclusion. Thank you for considering the revised version of our manuscript. Please see below our responses to individual comments.

1. Stats: One of the main results reported in the manuscript is male-female difference in exploratory and motor behavior, however, no direct statistical comparisons are made. Such comparisons are necessary to tell whether the differences exist. Otherwise, the ms. should not present any statement making sex-related comparisons. Please, pay careful attention to this point.

The reviewers highlight the need for direct statistics comparison of the male and female exploratory and motor behavior. As suggested, we included the comparison between males and females for WT and Cyfip1+/- animals. It was not possible to include the comparison for the Nlgn3y/- males and Nlgn3+/- females. Nlgn3 is an X-linked gene, and therefore the genotype of the males and females do not directly correspond to each other in this context. While the males lack Nlgn3 entirely, the females are heterozygous for Nlgn3, which introduces the gene dosage as an additional variable in addition to sex. We found that sex had no effect on distance travelled and time spent in the center of the open field for WT and Cyfip1+/- animals. However, there was a difference in the motor behavior depending on sex of the animals. We modified the text and figures to reflect this finding.

Details of the edits:

We added Extended Data 1-1 C to illustrate the effect of sex on distance travelled in the open field, and the details of the statistical test into the Statistical Table. We also included the description of the results in lines 258 - 262.

We added Extended Data 1-1 D to illustrate the effect of sex on time spent in the center of the open field, and the details of the statistical test into the Statistical Table. We also included the description of the results in lines 258 - 262..

We added Extended Data 2-1 C to illustrate the effect of sex on motor learning, and the details of the statistical test into the Statistical Table. We also included the description of the results in lines 297 - 301.

2. In the motor learning results, statistical comparisons between genotypes are missing. In addition, the authors report that WT male mice have a lower spine density in the motor cortex compared to previous reports. The ms. indicates that the lower spine density in the motor cortex reported here for WT male mice may be due to housing conditions. This interpretation implies that the observed increase of dendritic spines in the double-transgenic animals might result from a lower basal level in WT animals. To justify the statement that the reduced density of spines in male mice was caused by social environment, the spine density analysis should be carried out in mice housed with the same and other genotype (as for the transcriptome analysis). The authors should provide more rigorous evidence by analyzing spine density in SGH (single genotype housing) and MGH (multiple genotype housing) WT and transgenic animals. These analyses will clarify the effects of social environment on spine density in motor cortex of ASD animal models.

The reviewers point out that the statistical comparison between genotypes is missing. The details of the statistical analysis can be found in the Statistical Tables. We believe that the effect of genotype on the latency to fall as averaged across the different trials and days does not accurately reflect the ability to learn new motor routines hence the analysis of individual learning curves is included. To address the reviewer’s concern, we added a comment about the effect of genotype regardless of the day and trial.

The reviewers also suggest that the spine density analysis should be conducted in single genotype housed animals to investigate if the low spine density in the WT is a result of the effect of housing. While this would certainly be the ideal addition to the manuscript, it will not be possible to breed a new cohort of single genotype housed animals with the Thy-EGFP transgene and conduct the experiment within the 3 months timeframe and under the current restrictions relating to COVID-19 pandemic. Instead the mention of the social environment effect on the dendritic spines is removed from the results sections and instead discussed at the end of the manuscript.

Details of the edits:

Added a comment about the effect of genotype on latency to fall off rotarod in line 271-274.

Discussion of the possibility of social environment effect on dendritic spine density was added in lines 527 - 550.

3. We encourage the authors to explore the existence of a potential link between spine density and motor learning deficits in the transgenic mouse lines. In fact, while the heterozygote elimination of Nlg3 rescues Cyfip+/-, the same mutation appears to further enhance spine density compared to WT or single-transgenic. The authors are encouraged to analyze spine morphology and classify spines as filopodia, mushroom, stubby, etc. This will help to better understand whether there is a persistence of immature spines in the double transgenic, or whether those spines have the morphology of a mature (and functioning) spine.

The reviewers suggest that we investigate the link between spine density and motor learning. Although Yang et al. 2009 showed that acquisition of motor learning was associated with an increased formation of dendritic spines in the motor cortex, the necessity of spine formation for the acquisition of motor learning was never formally demonstrated (Yang et al. 2009). In our publication in 2019 showed despite lacking motor learning abilities, Cyfip1+/- mice showed unaltered formation of new spines in the motor cortex, indicating that the causal link between spine formation and motor learning is in fact not that direct (Bachmann et al. 2019). The link between expression of mRNA in specific brain region and the formation of dendritic spines or a behavioural performance is even more tenuous to draw. Therefore, we have decided from the start to consider the different levels of analysis (behaviour, synaptic and molecular) as separated and treated them as means to compare the different genotypes, sex and social condition. This point is now clarified in the manuscript.

Details of the edits:

Clarification about the purpose of the study added in lines 217- 218.

The reviewers also suggest that we classify the dendritic spines according to their morphology. While this would be an interesting information to add, we currently do not have the appropriate equipment to reliably gather this data. Classification of dendritic spines based on conventional light microscopy can lead to misclassification and misinterpretation. For reliable classification of dendritic spines based on morphology, we would require access to Stimulated Emission Depletion (STED) microscope, which we currently don’t have (Wijetunge et al. 2014).

4. We appreciate the remarkable effort in analyzing the transcriptome of those different ASD mouse models. However, the link of these results with motor learning impairment and spine density is missing. We understand that this is a complex task, but would advise testing the effects of different genotypes and housing conditions on spine density in the hippocampus (eg in CA1 neurons).

The reviewers suggest that in order to establish the connection between the motor learning/spine density and the transcriptome analysis, the effect of genotype and housing on the spine density in the hippocampus is established. The intention of the article was to extend the behavior and dendritic spine analysis present in the (Bachmann et al. 2019) to the Nlgn3y/-Cyfip1+/- double mutant animals. The RNA sequencing was added to further extend the analysis and the single genotype housing group was added to account for the possibility that the social environment might influence the transcriptome. This point is now clarified in the manuscript.

Another point to consider is that we are not confident that dendritic spines is a reliable measure. Considering lack of reproducibility of the decrease of dendritic spine density of the Cyfip1+/- males, the unexpectedly low levels of dendritic spines in the WT animals and a sex difference that has never been reported before, it is possible that there factors at play that have not been accounted for. In addition it is difficult to currently gather further data due to the COVID-19 related disruption. We included this point in the discussion.

Details of the edits:

Modified lines 69-80 in order to clarify the aims of the study.

5. Results need to be clarified: In the experiments presented in figs 5 and 6, the wild type animals from a different breeding line than the mutant animals and from the same breeding line as the mutant animals were used, respectively. The results section describes which ‘modules’ were up- and down-regulated. In the former experiment the modules are 8 and 17 and, in the latter - 9,10/14,16,18. From the description on the figs, we assume that they are different sets of genes. It is not clearly stated in the manuscript and the meaning of the difference between housing conditions that depend on the breeding line is not discussed.

The reviewers point out that the differences in gene identity between the different modules are not discussed. In the two RNA sequencing experiments, the content of the individual modules is indeed different. The modules networks are built each time based on the correlation matrix of the genes found in a particular dataset, so differences between datasets results in variable modules assignment. The difference in the membership of genes in the up- and down-regulated modules in the WT animals from the two different experiments indicated that there is a variability in the genes expression of which is dependent on the social environment. The conclusion from this two sets of experiments is however that there are differenced depending on the social environment, even if the identity of the genes involved might vary between different cohorts. We added comments in the results section about the gene identity in the different modules and we discuss this point.

Details of the edits:

Comment on the different gene identity between the two cohorts added in the results section, lines 466 - 468.

Discussion of the finding was included in the lines 551 - 560.

6. In the discussion, the authors should provide a more thorough explanation on why the interaction between Cyfip and Nlg3 may be important, especially considering the ability of Nlg3y/- to rescue Cyfip+/- motor learning in males and the inability of Cyfip+/- to rescue Nlg3y/- hyperactivity.

The reviewers request further clarification on the relationship between Neuroligin3 and Cyfip1. The rescue of the motor deficit in the Cyfip1+/- males by deleting Nlgn3 suggests the possibility that under physiological conditions Neuroligin3 inhibits Cyfip1. Therefore, when Nlgn3 is deleted, some of the trapped Cyfip1 is released and allowed to fulfill its function leading to restoration of the motor behavior. Another point to consider is that while complete deletion of Nlgn3 lead to restoration of motor learning in the Cyfip1+/- males, it is less likely that a mere reduction in the levels of Cyfip1 will lead to the correction of phenotype associated with a deletion of Nlgn3. The levels of Cyfip1 in the Cyfip1+/- males also varies depending on the brain region (Bachmann et al. 2019), raising the possibility that the reduction in Cyfip1 might be minimal in the circuits responsible for exploratory behavior. Discussion of these points is now included in the revised manuscript.

Details of the edits:

Discussion of the functional relationship between Neuroligin3 and Cyfip1 was included in lines 492 - 511.

Minor points:

7. According to the DMS-5, a deficit in cognitive flexibility is not per sè a symptom that might lead to the diagnosis of a disorder in the autistic spectrum. The two common symptoms of ASDs are social communication deficits and stereotyped behavior. Although stereotypies might be behavioral manifestations of impairments in cognitive flexibility, the latter term refers to a more complex phenotype that includes (but is not restricted to) action repetition, reversal-learning deficits in cue-outcome or action-outcome associations and deficits in adaptations of action strategies. I encourage the authors to use appropriate terms when referring to the ASD symptomatology.

The introduction was changed to reflect the DSM-5 more accurately, lines 30-32.

8. The nomenclature of the transgenic lines utilized in this manuscript somewhat confusing. The authors refer to the experimental group of Nlg3 heterozygous KO as Nlg3y/-, Nlg3+/- and Nlg3HET. I invite the authors to use the same nomenclature throughout the text and figures, unless justified. Moreover, the use of the nomenclature Nlg3y/-,Cyfip+/- in reference to the double-transgenic line is confusing, because of the use of commas (,) as punctuation elements in the text. To facilitate a clear reading and understanding of the results, I invite the authors to refer to the double-transgenic line as Nlg3y/-::Cyfip+/-, or other clearer nomenclature of their choice.

As Nlgn3 is an X-linked gene and therefore males can only have one copy of the gene, while the females can have two. The knockout males therefore lack the Nlgn3 on the X chromosome as well as Y chromosome, while the heterozygous females have a copy of Nlgn3 only on one of the X chromosomes. Therefore, the knockout males are marked as Nlgn3y/- while the heterozygous females are marked as Nlgn3+/-. Similarly, double mutant males are marked as Nlgn3y/-Cyfip1+/- and double mutant females are marked as Nlgn3+/-Cyfip1+/-. Any other nomenclature has been now removed from the manuscript.

9. Please, specify why the RNA sequencing is a technique “less prone to variability”? In what context? Why is it important here?

RNA sequencing is less prone to variability of samples within a condition in comparison to behaviour. We assume that the behaviour occurs downstream from RNA expression and as such there is more opportunity for other factors to affect it, increasing the variability within the groups. We have removed the mention of variability however, in order to emphasize that RNA sequencing represent another level of analysis and it is difficult to draw any link between it and behaviour.

10. Please, justify why they performed RNAseq of hippocampal tissue. Considering both the effects on behavior and dendritic spine, a more logical choice would have been the analysis of the motor cortex transcriptome.

Levels of CYFIP1 were shown to be reliably lowered in the hippocampi of Cyfip1+/- male mice (Bachmann et al. 2019) making it a suitable brain region for investigating the effect of Cyfip1 haploinsufficiency. On the other hand, the expression of Nlgn3 in the areas adjacent to the hippocampus that are likely to be included in the dissection of this area impacts on the phenotype in the Nlgn3 knockout mice. Specifically, learning motor routines is influenced by Nlgn3 expression in the nucleus accumbens (Rothwell et al. 2014), while social behaviour is affected by the deletion of Nlgn3 in the ventral tegmental area (Bariselli et al. 2018). Deleting Nlng3 in the hippocampus was also found to affect hippocampal dependent learning (Polepalli et al. 2017). Thus, the hippocampus is an area sensitive to Nlgn3 expression making it an interesting target for investigating the impact of Nlng3 deletion in shaping the transcription profile.

This explanation has now been included in the revised manuscript, lines 387 - 389. However, it is important to emphasize the point that while behaviour and dendritic spine density analysis aimed to extend previous results, the RNA sequencing is an additional assay that is not precisely linked with the other two.

11. The number of animals should be clearly stated in the results section and/or figures.

Numbers of animals were added into the results section.

12. The age of mice is described as P60 or older - the age range should be given more precisely.

A comment about the age of the animals was added in Methods lines 86 - 87.

13. Method, Animals - lack of an effect of the transgene on the behavioural outcomes was confirmed - this should be explained.

Every statistical analysis was repeated with transgenes as a factor. There were no significant differences between the animals with transgene and without. Added an explanation in lines 96 - 100.

14. Ultrasonic vocalisation - frequency of the vocalisations should be reported

- Please, refrain the use of “normal behavior” when it does not refer to a sample distribution (normal as in parametric). The authors are encouraged to adopt sentences like: “similar to controls” or “physiological”.

The frequency of vocalization is reported in Methods in line 1410 Mentions of normal behaviour have been removed.

5. Table 1-1 should better explain why in some cases (typically when performing one-way ANOVA) they assumed the normality of sample distributions, while in some other cases they did not and applied the non-parametric Kruskal-Wallis. What do they exactly mean by: “normality assumed based on histogram”?

The assumption of normality was verified using two methods: the visual method and suing the Shapiro-Wilk test. Shapiro-Wilk test is usually a reliable tool to detect deviation from normal distribution, however as the size of the sample increases the probability of Type 1 error increases (Ghasemi and Zahediasl 2012). Therefore, even though we have used the Shapiro-Wilk test as an aid, the assessment of the distribution of residuals was primarily based on the visual inspection of the histogram. When the histogram of residuals approached normal distribution, the normality assumption was assumed to be met. If the distribution of the residual deviated from the normal distribution a non-parametric test was used instead. An explanation added in lines 204 - 207.

16. Results, p. 10 - when the comparisons between mutants and wt mice are described, it is said for both Nlgn3 and double mutants: compared to their WT littermates, which suggests two WT groups but from the analysis it seems that there was only one WT group. It should be clarified.

Explanation added line 94 - 95.

17. Results, p.11 - ‘Unlike the male mice, all of the female mice showed a significant increase in the distance travelled between the first and second day of testing’ is inconsistent with what is shown in Fig 1D.

The sentence was corrected.

18. Throughout the manuscript there were many typos and grammar errors. The authors should consider carefully revising and correcting the manuscript.

Manuscript was revised.

References:

Bachmann, S.O., Sledziowska, M., Cross, E., Kalbassi, S., Waldron, S., Chen, F., Ranson, A., et al. (2019). Behavioral training rescues motor deficits in Cyfip1 haploinsufficiency mouse model of autism spectrum disorders. Translational Psychiatry 9:29.

Bariselli, S., Hörnberg, H., Prévost-solié, C., Musardo, S., Hatstatt-burklé, L., Scheiffele, P. and Bellone, C. (2018). Role of VTA dopamine neurons and neuroligin 3 in sociability traits related to nonfamiliar conspecific interaction. Nature Communications 9.

Ghasemi, A. and Zahediasl, S. (2012). Normality tests for statistical analysis: a guide for non-statisticians. International journal of endocrinology and metabolism 10:486-489.

Polepalli, J.S., Wu, H., Goswami, D., Halpern, C.H., Südhof, T.C. and Malenka, R.C. (2017). Modulation of excitation on parvalbumin interneurons by neuroligin-3 regulates the hippocampal network. 20.

Rothwell, P.E., Fuccillo, M. V., Maxeiner, S., Hayton, S.J., Gokce, O., Lim, B.K., Fowler, S.C., et al. (2014). Autism-associated neuroligin-3 mutations commonly impair striatal circuits to boost repetitive behaviors. Cell 158:198-212.

Wijetunge, L.S., Angibaud, J., Frick, A., Kind, P.C. and Na, U.V. (2014). Stimulated Emission Depletion (STED) Microscopy Reveals Nanoscale Defects in the Developmental Trajectory of Dendritic Spine Morphogenesis in a Mouse Model of Fragile X Syndrome. 34:6405-6412.

Yang, G., Pan, F. and Gan, W.B. (2009). Stably maintained dendritic spines are associated with lifelong memories. Nature 462:920-924.
