# Supplementary material for: Complex Interactions between Genes and Social Environment Cause Phenotypes Associated with Autism Spectrum Disorders in Mice
Source: eNeuro. 2020 Aug 5;7(4):ENEURO.0124-20.2020. doi: 10.1523/ENEURO.0124-20.2020 (PMC7418534; doi:10.1523/ENEURO.0124-20.2020)
Supplement: Figure 1-2 — Statistical table. Download Figure 1-2, DOCX file. [file enu-eN-NWR-0124-20-s01.docx]

Table 1-1 Details of statistical analyses.

| **Figure** | **Normality** | **Equality of variances** | **Group differences** | **Sample size** |
| --- | --- | --- | --- | --- |
| Figure 1 A | Shapiro-Wilk test  *W* = 0.99  *P* = 0.91 | No heteroscedasticity | One-way ANOVA, main effect of genotype: *F*(3, 65) = 6.55 *P* < 0.001  Tukey HSD:  *Cyfip1^+/-^* vs *Nlgn3^y/-^* *t*(1, 65) = -2.46, *P* = 0.08  *Cyfip1^+/-^* vs *Nlgn3^y/-^Cyfip1^+/--^*  *t*(1, 65) = -2.70, *P* < 0.05  *Cyfip1^+/-^* vs *WT* *t*(1, 65) = 0.57, *P* = 0.94  *Nlgn3^y/-^* vs *Nlgn3^y/-^Cyfip1^+/-^ t*(1, 65) = -0.66, *P* = 0.91  *Nlgn3^y/-^* vs *WT* *t*(1, 65) = 3.47, *P* < 0.01  *Nlgn3^y/-^Cyfip1^+/-^* vs *WT* *t*(1, 65) = 3.53, *P* < 0.01 | *WT* n = 18  *Nlgn3^y/-^* n = 24  *Cyfip1^+/-^* n= 12  *Nlgn3^y/-^Cyfip1^+/-^* n = 12 |
| Figure 1 B | Shapiro-Wilk test  *W* = 0.99  *P* = 0.53 | No heteroscedasticity | Mix Model ANOVA, main effect of genotype: *F(3, 62)* = 6.45, *P <* 0.001, main effect of day: *F(1, 62)* = 35.54, *P* < 0 .001, interaction of the effect of genotype and day: *F(3, 62)* = 2.41, *P* = 0.08  Tukey HSD:  *WT* Day 1 vs *WT* Day 2 *t*(1, 62) = 5.29, *P* < 0.001  *Nlgn3^y/-^* Day 1 vs *Nlgn3^y/-^* Day 2 *t*(1, 62) = 2.21, *P* = 0.42  *Cyfip1^+/-^* Day 1 vs *Cyfip1^+/-^* Day 2 *t*(1, 62) = 1.99, *P* = 0.50  *Nlgn3^y/-^Cyfip1^+/-^* Day 1 vs *Nlgn3^y/-^Cyfip1^+/-^* Day 2 *t*(1, 62) = 2.52, *P* = 0.21  *WT* vs *Cyfip1^+/-^ t*(1, 62) = 0.42, *P* = 0.98  *WT* vs *Nlgn3^y/-^ t*(1, 62) = -2.70, *P* < 0.05  *WT* vs *Nlgn3^y/-^Cyfip1^+/-^ t*(1, 62) = -3.53, *P* < 0.01  *Cyfip1^+/-^* vs *Nlgn3^y/-^ t*(1, 62) = -2.82, *P* < 0.05  *Cyfip1^+/-^* vs *Nlgn3^y/-^Cyfip1^+/-^ t*(1, 62) = -3.61, *P* < 0.01  *Nlgn3^y/-^* vs *Nlgn3^y/-^Cyfip1^+/-^ t*(1, 62) = -1.35, *P* = 0.54 | *WT* n = 18  *Nlgn3^y/-^* n = 24  *Cyfip1^+/-^* n= 12  *Nlgn3^y/-^Cyfip1^+/-^* n = 12 |
| Figure 1 C | Shapiro-Wilk test  *W* = 0.97  *P* = 0.12 | No heteroscedasticity | One-way ANOVA, main effect of genotype: *F(3, 59)* = 4.64, *P <* 0.01  Tukey HSD:  *Cyfip1^+/-^* vs *Nlgn3^+/-^* *t*(1, 59) = -3.34, *P* < 0.01  *Cyfip1^+/-^* vs *Nlgn3^+/-^Cyfip1^+/--^*  *t*(1, 59) = -1.05, *P* = 0.72  *Cyfip1^+/-^* vs *WT* *t*(1, 59) = *-*1.20, *P* = 0.63  *Nlgn3^+/-^* vs *Nlgn3^+/-^Cyfip1^+/-^ t*(1, 59) = 2.66, *P* <0.05  *Nlgn3^+/-^* vs *WT* *t*(1, 59) = 2.42, *P* = 0.08  *Nlgn3^+/-^Cyfip1^+/-^* vs *WT* *t*(1, 59) = -0.19, *P* = 1.00 | *WT* n = 16  *Nlgn3^+/-^* n = 20  *Cyfip1^+/-^* n= 10  *Nlgn3^+/-^Cyfip1^+/-^* n = 17 |
| Figure 1 D | Shapiro-Wilk test  *W* = 0.99  *P* = 0.22 | No heteroscedasticity | Mix Model ANOVA, main effect of genotype: *F(3, 59)* = 4.51, *P <* 0.01,main effect of day: *F(1, 59)* = 92.13, *P* = <0.001, interaction of the effect of genotype and day: *F(3, 59)* = 0.56, *P* = 0.65.  Tukey HSD:  *WT* Day 1 vs *WT* Day 2 *t*(1, 59) = 5.61, *P* < 0.001  *Nlgn3^+/-^* Day 1 vs *Nlgn3^+/-^* Day 2 *t*(1, 59) = 4.42, *P* < 0.001  *Cyfip1^+/-^* Day 1 vs *Cyfip1^+/-^* Day 2 *t*(1, 59) = 3.83, *P* < 0.01  *Nlgn3^+/-^ Cyfip1^+/-^* Day 1 vs *Nlgn3^+/-^Cyfip1^+/-^* Day 2 *t*(1, 59) = 5.30, *P* < 0.001  *WT* vs *Cyfip1^+/-^ t*(1, 59) = 1.54, *P* = 0.42  *WT* vs *Nlgn3^+/-^ t*(1, 59) = -2.10, *P* = 0.16  *WT* vs *Nlgn3^+/-^Cyfip1^+/-^ t*(1, 59) = 0.36, *P* = 0.98  *Cyfip1^+/-^* vs *Nlgn3^+/-^ t*(1, 59) = -3.42, *P* < 0.01  *Cyfip1^+/-^* vs *Nlgn3^+/-^Cyfip1^+/-^ t*(1, 59) = -1.24, *P* = 0.60  *Nlgn3^+/-^* vs *Nlgn3^+/-^Cyfip1^+/-^ t*(1, 59) = 2.52, *P* = 0.07 | *WT* n = 16  *Nlgn3^+/-^* n = 20  *Cyfip1^+/-^* n= 10  *Nlgn3^+/-^Cyfip1^+/-^* n = 17 |
| Figure 2 A | Shapiro-Wilk test  *W* = 0.99  *P* < 0.001 | Heteroscedasticity | Non-parametric Mix Model ANOVA, main effect of genotype: *F(3, 53)* = 1.90, *P* = 0.13 ,main effect of day: *F(1, 1577)* = 57.36, *P* < 0.001, main effect of trial: *F(9, 1577)* = 2.68, *P* <0.01, interaction of the effect of genotype and day: *F(3, 1577)* = 4.61, *P <* 0.01.  Simple effects:  *WT*  Day1 vs Day 2: *t*(1, 16) = 2.12, *P* < 0.05  Day1 vs Day 3: *t*(1, 16) = 6.28, *P* < 0.001  Day2 vs Day 3: *t*(1, 16) = 2.86, *P* < 0.05  *Nlgn3^y/-^*  Day1 vs Day 2: *t*(1, 15) = 6.16, *P* < 0.001  Day1 vs Day 3: *t*(1, 15) = 9.14, *P* < 0.001  Day2 vs Day 3: *t*(1, 15) = 3.02, *P* < 0.05  *Cyfip1^+/-^*  Day1 vs Day 2: *t*(1, 11) = 1.10, *P* = 0.50  Day1 vs Day 3: *t*(1, 11) = 1.54, *P* = 0.39  Day2 vs Day 3: *t*(1, 11) = 1.11, *P* = 0.50  *Nlgn3^y/-^Cyfip1^+/-^*  Day1 vs Day 2: *t*(1, 11) = 5.05, *P* < 0.001  Day1 vs Day 3: *t*(1, 11) = 4.69, *P* < 0.001  Day2 vs Day 3: *t*(1, 11) = 1.73, *P* = 0.06 | *WT* n = 17  *Nlgn3^y/-^* n = 16  *Cyfip1^+/-^* n= 12  *Nlgn3^y/-^Cyfip1^+/-^* n = 12 |
| Figure 2 B | Shapiro-Wilk test  *W* = 0.98  *P* < 0.001 | Heteroscedasticity | Non-parametric Mix Model ANOVA, main effect of genotype: *F(3, 50)* = 2.88, *P* = 0.73, main effect of day: *F(1,* 1490*)* = 66.39, *P* < 0.001, main effect of trial: *F(9,* 1490*)* = 18.84, *P* <0.001, interaction of the effect of genotype and day: *F(3, 1577)* = 4.45, *P =* 0.13.  Simple effects:  *WT*  Day1 vs Day 2: *t*(1, 12) = 5.09, *P* < 0.01  Day1 vs Day 3: *t*(1, 12) = 5.85, *P* < 0.01  Day2 vs Day 3: *t*(1, 12) = 3.47, *P* = 0.07  *Nlgn3^+/-^*  Day1 vs Day 2: *t*(1, 12) = 6.15, *P* < 0.01  Day1 vs Day 3: *t*(1, 12) = 8.06, *P* < 0.001  Day2 vs Day 3: *t*(1, 12) = 4.23, *P* < 0.05  *Cyfip1^+/-^*  Day1 vs Day 2: *t*(1, 13) = 3.73 *P* = 0.05  Day1 vs Day 3: *t*(1, 13) = 5.95, *P* < 0.01  Day2 vs Day 3: *t*(1, 13) = 2.21, *P* = 0.29  *Nlgn3^y/-^Cyfip1^+/-^*  Day1 vs Day 2: *t*(1, 13) = 7.34 *P* < 0.001  Day1 vs Day 3: *t*(1, 13) = 7.41, *P* < 0.011  Day2 vs Day 3: *t*(1, 13) = 1.05, *P* = 0.74 | *WT* n = 13  *Nlgn3^+/-^* n = 13  *Cyfip1^+/-^* n= 14  *Nlgn3^+/-^Cyfip1^+/-^* n = 14 |
| Figure 3 A | Shapiro-Wilk test  *W* = 0.87  *P* < 0.001 | Heteroscedasticity | Non-parametric Mix Model ANOVA, main effect of genotype: *F(3, 50)* = 0.68, *P* = 0.56, main effect of trial: *F(2, 100)* = 41.73, *P* < 0.001, interaction of the effect of genotype and trial: *F(6, 100)* = 0.57, *P =* 0.73.  Simple effects:  *WT*  C1 vs C2: *t*(1, 13) = 3.07, *P* = 0.18  C1 vs S1: *t*(1, 13) = 2.70 *P* = 0.27  C1 vs S2: *t*(1, 13) = 1.60, *P* = 0.68  C2 vs S1: *t*(1, 13) = 4.60, *P* < 0.05  C2 vs S2: *t*(1, 13) = 1.14, *P* = 0.85  S1 vs S2: *t*(1, 13) = 3.68, *P* = 0.09  *Nlgn3^y/-^*  C1 vs C2: *t*(1, 15) = 11.8 *P* < 0.001  C1 vs S1: *t*(1, 15) = 2.27 *P* = 0.41  C1 vs S2: *t*(1, 15) = 0.15, *P* = 1.00  C2 vs S1: *t*(1, 15) = 5.91, *P* < 0.01  C2 vs S2: *t*(1, 15) = 2.56, *P* = 0.31  S1 vs S2: *t*(1, 15) = 1.49, *P* = 0.72  *Cyfip1^+/-^*  C1 vs C2: *t*(1, 11) = 4.04 *P* = 0.06  C1 vs S1: *t*(1, 11) = 1.08 *P* = 0.87  C1 vs S2: *t*(1, 11) = 1.30, *P* = 0.80  C2 vs S1: *t*(1, 11) = 8.74, *P* < 0.001  C2 vs S2: *t*(1, 11) = 4.69, *P* < 0.05  S1 vs S2: *t*(1, 11) = 3.37, *P* = 0.14  *Nlgn3^y/-^Cyfip1^+/-^*  C1 vs C2: *t*(1, 12) = 2.29 *P* = 0.41  C1 vs S1: *t*(1, 12) = 3.87 *P* = 0.08  C1 vs S2: *t*(1, 12) = 1.10, *P* = 0.86  C2 vs S1: *t*(1, 12) = 4.40, *P* < 0.05  C2 vs S2: *t*(1, 12) = 4.71, *P* < 0.05  S1 vs S2: *t*(1, 12) = 2.45, *P* = 0.35 | *WT* n = 14  *Nlgn3^y/-^* n = 16  *Cyfip1^+/-^* n= 12  *Nlgn3^y/-^* *Cyfip1^+/-^* n = 12 |
| Figure 3 B | Shapiro-Wilk test  *W* = 0.89  *P* < 0.001 | Heteroscedasticity | Kruskal-Wallis: *χ^2^(3, 50)* = 1.51, *P* = 0.68 | *WT* n = 14  *Nlgn3^y/-^* n = 16  *Cyfip1^+/-^* n= 12  *Nlgn3^y/-^* *Cyfip1^+/-^* n = 12 |
| Figure 3 C | Shapiro-Wilk test  *W* = 0.90  *P* < 0.001 | Heteroscedasticity | Non-parametric Mix Model ANOVA, main effect of genotype: *F(3, 50)* = 0.81, *P* = 0.48,main effect of trial: *F(2, 100)* = 41.07, *P* < 0.001, interaction of the effect of genotype and trial: *F(6, 100)* = 1.26 *P =* 0.28  Simple effects:  *WT*  C1 vs C2: *t*(1, 12) = 2.43, *P* = 0.36  C1 vs S1: *t*(1, 12) = 8.31 *P* < 0.001  C1 vs S2: *t*(1, 12) = 0.41, *P* = 0.99  C2 vs S1: *t*(1, 12) = 10.79, *P* < 0.001  C2 vs S2: *t*(1, 12) = 1.57, *P* = 0.69  S1 vs S2: *t*(1, 12) = 3.11, *P* = 0.18  *Nlgn3^+/-^*  C1 vs C2: *t*(1, 12) = 2.46, *P* = 0.35  C1 vs S1: *t*(1, 12) = 2.62 *P* = 0.30  C1 vs S2: *t*(1, 12) = 1.01, *P* = 0.89  C2 vs S1: *t*(1, 12) = 5.50, *P* = 0.01  C2 vs S2: *t*(1, 12) = 1.73, *P* = 0.62  S1 vs S2: *t*(1, 12) = 4.79, *P* < 0.05  *Cyfip1^+/-^*  C1 vs C2: *t*(1, 13) = 3.85, *P* = 0.07  C1 vs S1: *t*(1, 13) = 1.99 *P* = 0.52  C1 vs S2: *t*(1, 13) = 0.42, *P* = 0.99  C2 vs S1: *t*(1, 13) = 4.82, *P* < 0.05  C2 vs S2: *t*(1, 13) = 3.27, *P* = 0.15  S1 vs S2: *t*(1, 13) = 1.12, *P* = 0.86  *Nlgn3^y/-^Cyfip1^+/-^*  C1 vs C2: *t*(1, 13) = 2.23, *P* = 0.42  C1 vs S1: *t*(1, 13) = 5.95 *P* < 0.01  C1 vs S2: *t*(1, 13) = 0.97, *P* = 0.90  C2 vs S1: *t*(1, 13) = 5.44, *P* < 0.01  C2 vs S2: *t*(1, 13) = 2.38, *P* = 0.37  S1 vs S2: *t*(1, 13) = 6.39, *P* < 0.01 | *WT* n = 13  *Nlgn3^+/-^* n = 13  *Cyfip1^+/-^* n= 14  *Nlgn3^+/-^Cyfip1^+/-^* n = 14 |
| Figure 3 D | Shapiro-Wilk test  *W* = 0.97  *P* = 0.21 | Heteroscedasticity | One-way ANOVA, main effect of genotype: *F(3, 50)* = 2.92, *P* <0.05 | *WT* n = 13  *Nlgn3^+/-^* n = 13  *Cyfip1^+/-^* n= 14  *Nlgn3^+/-^Cyfip1^+/-^* n = 14 |
| Figure 3 E | Shapiro-Wilk test  *W* = 0.94  *P* < 0.01 | Heteroscedasticity | Kruskal-Wallis, main effect of genotype: *χ^2^ (3, 52)* = 2.04 *P* = 0.56 | *WT* n = 17  *Nlgn3^y/-^* n = 16  *Cyfip1^+/-^* n= 12  *Nlgn3^y/-^* *Cyfip1^+/-^* n = 12 |
| Figure 3 F | Shapiro-Wilk test  *W* = 0.94  *P* < 0.01 | Heteroscedasticity | Kruskal-Wallis, main effect of genotype: *χ^2^ (3, 52)* = 1.98 *P* = 0.57 | *WT* n = 17  *Nlgn3^y/-^* n = 16  *Cyfip1^+/-^* n= 12  *Nlgn3^y/-^* *Cyfip1^+/-^* n = 12 |
| Figure 3 G | Shapiro-Wilk test  *W* = 0.99  *P* = 0.46 | Heteroscedasticity | One-way ANOVA, main effect of genotype: *F(3, 53)* = 0.08 *P* = 0.97 | *WT* n = 17  *Nlgn3^y/-^* n = 16  *Cyfip1^+/-^* n= 12  *Nlgn3^y/-^* *Cyfip1^+/-^* n = 12 |
| Figure 4 A | Shapiro-Wilk test  *W* = 0.97  *P* < 0.01 | No heteroscedasticity | Kruskal-Wallis, *χ^2^(3,153)* = 15.64, *P <* 0.01  Dunn’s test with Bonferroni correction:  *WT* vs *Nlgn3^y/-^ z*(1, 153) = -0.11, *P* = 1.00  *WT* vs *Cyfip1^+/-^ z*(1, 153) = -1.98, *P* = 0.14  *WT* vs *Nlgn3^y/-^Cyfip1^+/-^ z(*1, 153) = -3.41, *P* < 0.01  *Nlgn3^y/-^* vs *Cyfip1^+/-^ z*(1, 153) = *-*1.84, *P* = 0.20  *Nlgn3^y/-^* vs *Nlgn3^y/-^Cyfip1^+/-^ z*(1, 153) = -3.27, *P* < 0.01  *Cyfip1^+/-^* vs *Nlgn3^y/-^Cyfip1^+/-^ z*(1, 153) = 1.56, *P* = 0.36 | *WT* n = 40  *Nlgn3^y/-^* n = 39  *Cyfip1^+/-^* n = 43  *Nlgn3^y/-^Cyfip1^+/-^* n = 35 |
| Figure 4 A | Shapiro-Wilk test  *W* = 0.54  *P* = 0.65 | No heteroscedasticity | One-way ANOVA, main effect of genotype: *F(3,121)* = 1.16, *P* = 0.33 | *WT* n = 41  *Nlgn3^y/-^* n = 36  *Cyfip1^+/-^* n = 28  *Nlgn3^y/-^ Cyfip1^+/-^* n = 20 |
| Figure 4 B | Shapiro-Wilk test  *W* = 0.95  *P* < 0.001 | No heteroscedasticity | Kruskal-Wallis, main effect of genotype: *χ^2^(3, 176)* = 0.17, *P* = 0.98 | *WT* n = 47  *Nlgn3^+/-^* n = 43  *Cyfip1^+/-^* n = 46  *Nlgn3^+/-^Cyfip1^+/-^* n = 44 |
| Figure 4 B | Shapiro-Wilk test  *W* = 0.94  *P* < 0.001 | No heteroscedasticity | Kruskal-Wallis, main effect of genotype: *χ^2^ (3, 158)* = 5.25, *P* = 0.15 | *WT* n = 40  *Nlgn3^+/-^* n = 42  *Cyfip1^+/-^* n = 45  *Nlgn3^+/-^Cyfip1^+/-^* n = 35 |
| Extended Data Figure 1-1 A | Shapiro-Wilk test  *W* = 0.97  *P* < 0.01 | No heteroscedasticity | Non-parametric Mix Model ANOVA, main effect of genotype: *F(3, 62)* = 0.54, *P* = 0.63, main effect of day: *F(1, 62)* = 2.69, *P* = 0.10, interaction of the effect of genotype and day: *F(3, 62)* = 0.32, *P* = 0.81 | *WT* n = 18  *Nlgn3^y/-^* n = 24  *Cyfip1^+/-^* n= 12  *Nlgn3^y/-^Cyfip1^+/-^* n = 12 |
| Extended Data Figure 1-1 B | Shapiro-Wilk test  *W* = 0.99  *P* = 0.27 | No heteroscedasticity | Mix Model ANOVA, main effect of genotype: *F(3, 59)* = 0.13, *P* = 0.94, main effect of day: *F(1, 59)* = 0.52, *P* = 0.47,interaction of the effect of genotype and day: *F(3, 59)* = 0.84, *P* = 0.48 | *WT* n = 16  *Nlgn3^+/-^* n = 20  *Cyfip1^+/-^* n= 10  *Nlgn3^+/-^Cyfip1^+/-^* n = 17 |
| Extended Data Figure 1-1 C | Shapiro-Wilk test  *W* = 0.99  *P* = 0.72 | No heteroscedasticity | Two-way ANOVA, main effect of genotype: *F(1, 52)* = 0.16, *P* = 0.69, main effect of sex: *F(1, 52)* = 0.26, *P* = 0.61, interaction between the effect of genotype and sex: *F(1, 52)* = 1.82, *P* = 0.18. | *Males:*  *WT* n = 18  *Cyfip1^+/-^* n= 12  *Females:*  *WT* n = 16  *Cyfip1^+/-^* n= 10 |
| Extended Data Figure 1-1 D | Shapiro-Wilk test  *W* = 0.91  *P* < 0.001 | No heteroscedasticity | Scheirer–Ray–Hare test, main effect of genotype: *H(1, 52)* = 1.48, *P* = 0.22, main effect of sex: *H(1, 52)* = 0.18 , *P* = 0.67, interaction between the effect of genotype and sex: *H(1, 52)* = 0.01, *P* = 0.93. | *Males:*  *WT* n = 18  *Cyfip1^+/-^* n= 12  *Females:*  *WT* n = 16  *Cyfip1^+/-^* n= 10 |
| Extended Data Figure 1-1 E | Shapiro-Wilk test  *W* = 0.99  *P* = 0.80 | No heteroscedasticity | Two-way ANOVA, main effect of genotype: *F(1, 74)* = 17.19, *P <* 0.001, main effect of sex: *F(1, 74)* = 0.01, *P =* 0.93, interaction between the effect of genotype and sex: *F(1, 74)* = 0.38, *P* = 0.54. | *Males:*  *WT* n = 18  *Nlgn3^y/-^* n = 24  *Females:*  *WT* n = 16  *Nlgn3^+/-^* n = 20 |
| Extended Data Figure 1-1 F | Shapiro-Wilk test  *W* = 0.96  *P* = 0.02 | No heteroscedasticity | Scheirer–Ray–Hare test, main effect of genotype: *H(1, 74)* = 0.01, *P* = 0.98, main effect of sex: *H(1, 74)* = 0.25 , *P* = 0.62, interaction between the effect of genotype and sex: *H(1, 74)* = 1.03, *P* = 0.31. | *Males:*  *WT* n = 18  *Nlgn3^y/-^* n = 24  *Females:*  *WT* n = 16  *Nlgn3^+/-^* n = 20 |
| Extended Data Figure 2-1 C | Shapiro-Wilk test  *W* = 0.98  *P* < 0.001 | No heteroscedasticity | Non-parametric Mix Model ANOVA, main effect of genotype: *F(1, 52)* = 1.01, *P* = 0.32, main effect of day: *F(1, 52)* = 27.26, *P* < 0.001, main effect of sex: *F(1, 52)* = 4.88, *P <* 0.05. | *Males:*  *WT* n = 17  *Cyfip1^+/-^* n= 12  *Females:*  *WT* n = 13  *Cyfip1^+/-^* n= 14 |
| Extended Data Figure 2-1 D | Shapiro-Wilk test  *W* = 0.98  *P* < 0.001 | No heteroscedasticity | Non-parametric Mix Model ANOVA, main effect of genotype: *F(1, 55)* = 2.78, *P* = 0.10 main effect of day: *F(1, 55)* = 97.01, *P* < 0.001, main effect of sex: *F(1, 55)* = 0.13, *P =* 0.72. | *Males:*  *WT* n = 17  *Nlgn3^y/-^* n = 16  *Females:*  *WT* n = 13  *Nlgn3^+/-^* n = 13 |
| Extended Data Figure 3-1 | Shapiro-Wilk test  *W* = 0.95  *P* < 0.001 | Heteroscedasticity | Scheirer–Ray–Hare, main effect of sex: *H*(1,326) = 98.97, *P* < 0.001, main effect of genotype: *H*(1,326) = 3.99, P < 0.05, sex and genotype interaction: *H*(1,326) = 0.45, *P* = 0.50. | *Males:*  *WT* n = 81  *Cyfip1^HET^* n = 71  Females:  *WT* n = 87  *Cyfip1^HET^* n = 91 |
